# Supplementary material for: Epigenetic and evolutionary features of ape subterminal heterochromatin
Source: Genome Res. 2026 Jan;36(1):38–49. doi: 10.1101/gr.280987.125 (PMC12758386; doi:10.1101/gr.280987.125)
Supplement: Supplement 1 [file Supplemental_Figs_S1-S35_and_legends.pdf]

Epigenetic and evolutionary features of ape subterminal heterochromatin

Yoo et al.

Table of Contents

Supplemental figures S1-S34 2

Supplemental data legend 34

Supplemental table legends S1-S7 35

Supplemental figures S1-S34

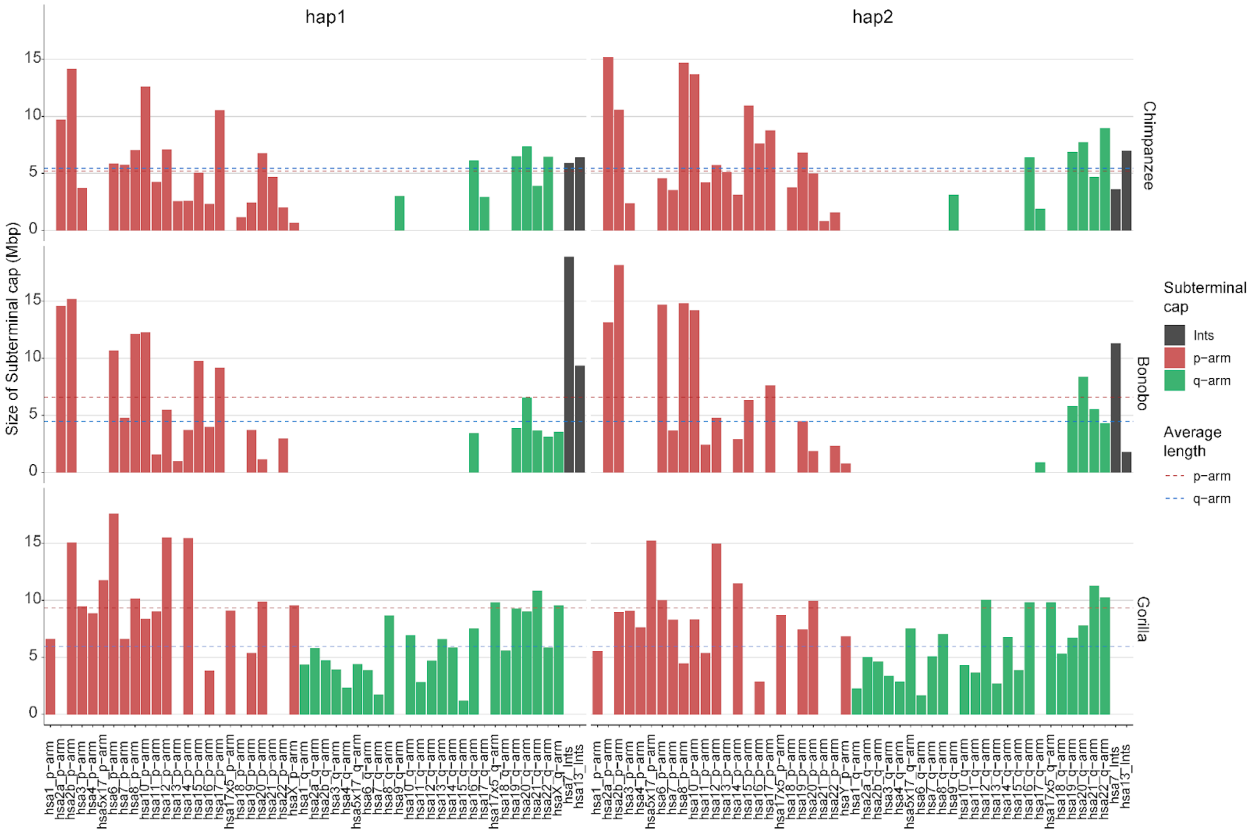

Supplemental Figure S1. Subterminal caps of African great ape genomes. Size of the satellite array in Mbp is summarized for each subterminal cap, with the position indicated by different colors. Dotted line indicates the average size of the p- and q-arm subterminal satellite arrays.

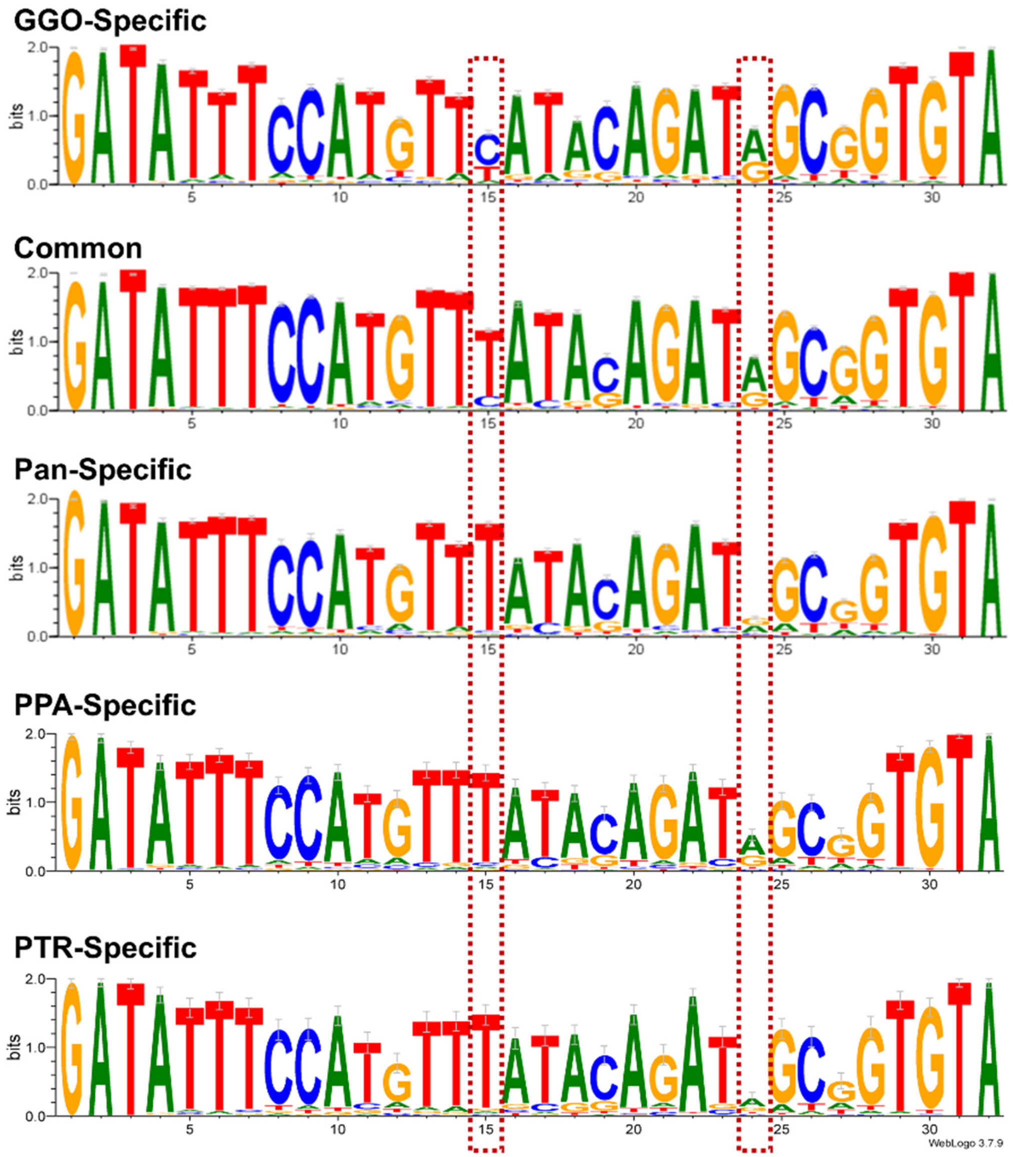

Supplemental Figure S2. Profile of pCht satellite repeats. The number of pCht variant types used for the profiles were as follows: GGO-specific (n=4,724), common (n=8,873), *Pan*-specific (n=24,204), PPA-specific (n=549), and PTR-specific (n=326).

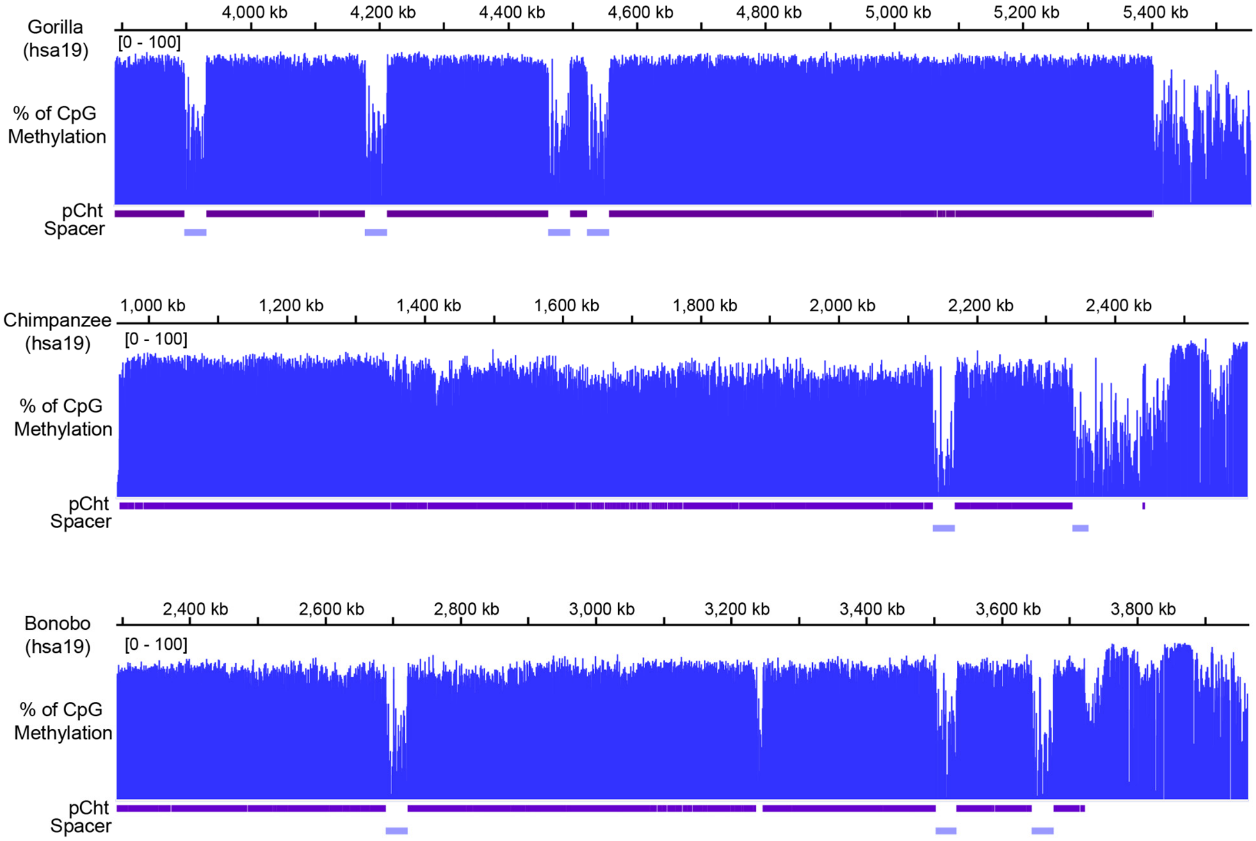

Supplemental Figure S3. 5mC methylation status of the hsa19 p-arm subterminal caps of gorilla, chimpanzee and bonobo, composed of hypomethylated pockets of spacer segmental duplications (SDs). Below the methylation tracks are the locations of pCht satellite arrays and SD spacers in purple and light blue.

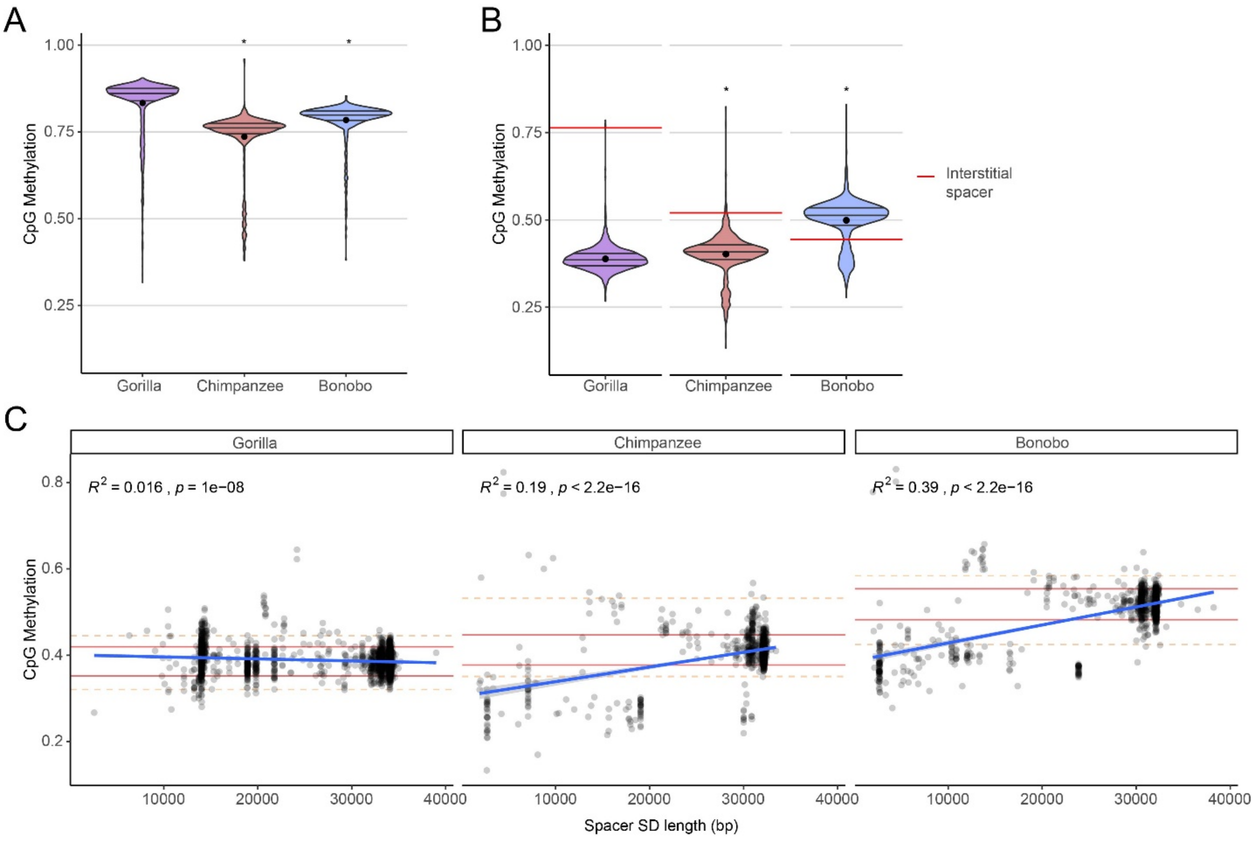

Supplemental Figure S4. Comparison of CpG methylation status at subterminal regions.
(A) Distribution of average 5mC methylation of non-interrupted pCht satellite arrays.
(B) Distribution of the average 5mC methylation of subterminal SD spacers. The average of 5mC methylation of subterminal SD spacers outside of subterminal satellite arrays is indicated by red. For a and b, the statistically significant difference compared to bonobo was tested by two-sided Wilcox test and the * indicates significant difference with p<2.2e-16. **(C)** Distribution of average 5mC methylation of total subterminal SD spacers with different lengths. The blue line shows the linear regression line whose correlation coefficient (R^2) and p-value are indicated on top of each plot. Red lines indicate top/bottom 5% quantile observed for full-length SD spacers (>32 kbp and >34 kbp for *Pan* lineage and gorilla, respectively) and the orange dotted lines indicate maximum/minimum observed for the full-length SD spacers.

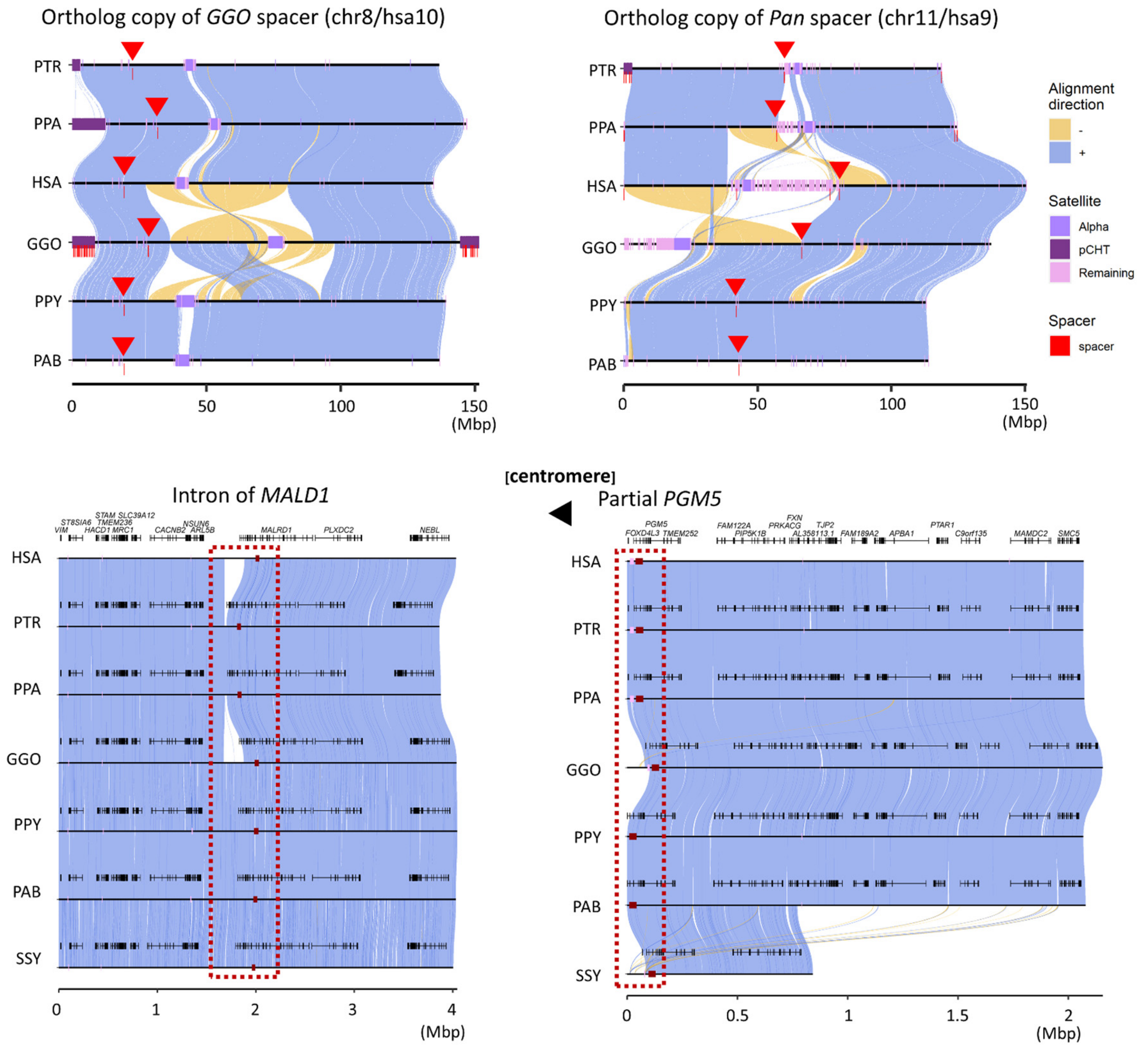

Supplemental Figure S5. Location of the seed sequence of *Pan* lineage and gorilla subterminal SDs identified using orthologs. The whole chromosome alignment view (top) shows syntenic alignment of hsa10 and hsa9; human (HSA), chimpanzee (PTR), bonobo (PPA), gorilla (GGO), Bornean and Sumatran orangutan (PPY and PAB), and siamang (SSY). Blue and yellow indicate syntenic and inverted alignments, respectively. Satellite tracks are shown for each of the species, including alpha, pCht subterminal, and the remaining satellites. The red track below the satellites and red triangle above the tracks indicate the position of SD spacers. Below this, the 4 and 2 Mbp zoomed-in views (bottom) illustrate syntenic genes and structural variants near the core sequence of the SD spacers.

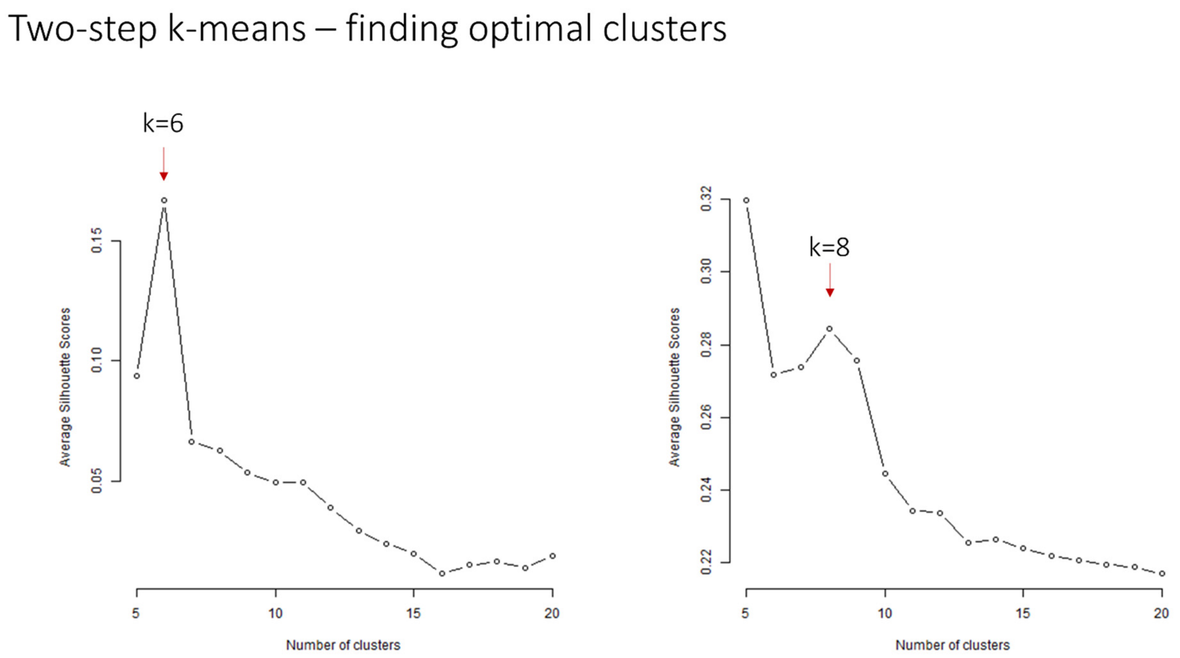

Supplemental Figure S6. Silhouette score of the two-step (left - first and right - second) *k*-means clustering to find optimal number of translocating groups and 20 kbp block types.

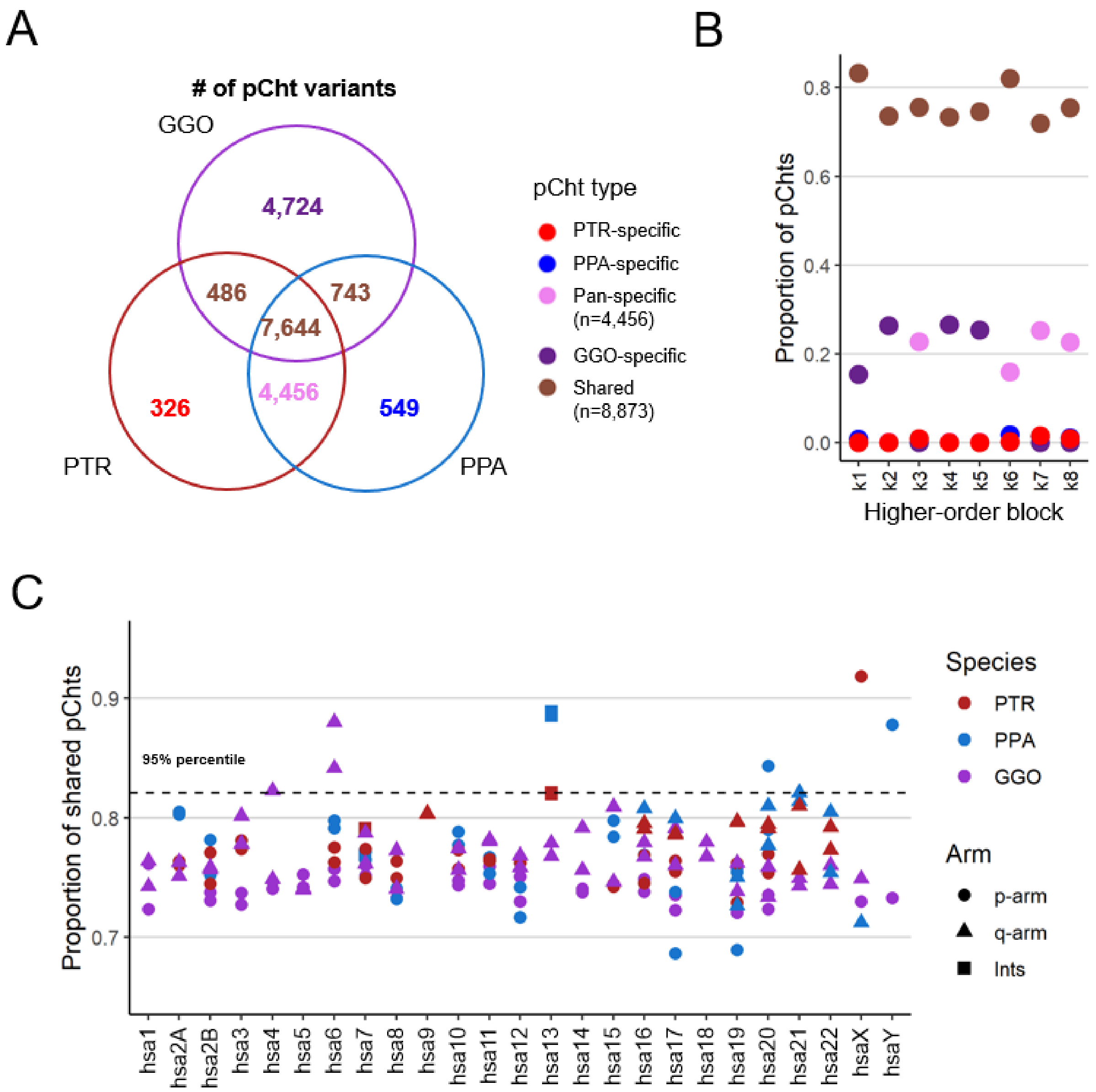

Supplemental Figure S7. Composition of pCht variant types in the African great ape genomes. (A) Venn diagram of species-specific and shared pCht variants. **(B)** Lineage-specific pCht composition in each higher-order block (k1-8). **(C)** Proportion of the shared pCht variants in each subterminal cap.

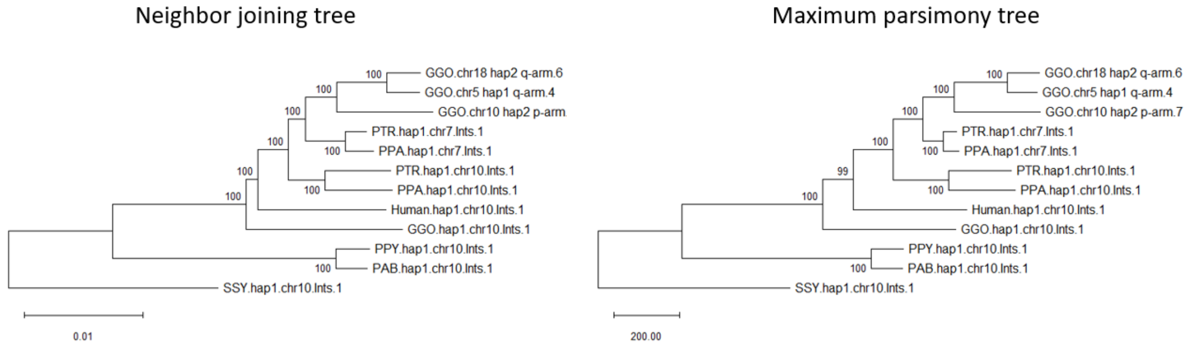

Supplemental Figure S8. Neighbor-joining and maximum parsimony phylogenetic trees of subset of gorilla spacers. Internal nodes are labelled by bootstrap score supporting the topology. Acronyms of terminal nodes indicate haplotype (hap1 or hap2) and species: chimpanzee (PTR), bonobo (PPA), gorilla (GGO), Sumatran orangutan (PAB), Bornean orangutan (PPY), and siamang (SSY).

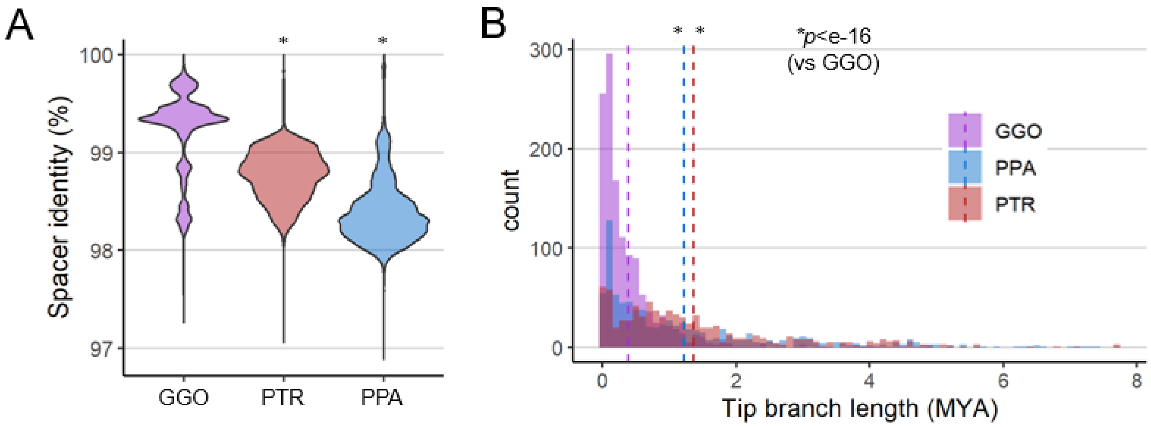

Supplemental Figure S9. Divergence of the SD spacers. (A) Pairwise identity distribution among the SD spacers in three African great ape species. **(B)** Histogram of the tip branch length estimated from maximum likelihood. The mean values are indicated as a dotted line. “*” in panels A and B, above PTR and PPA, indicates significant difference compared to GGO (p<e-16, two-sided Wilcoxon test).

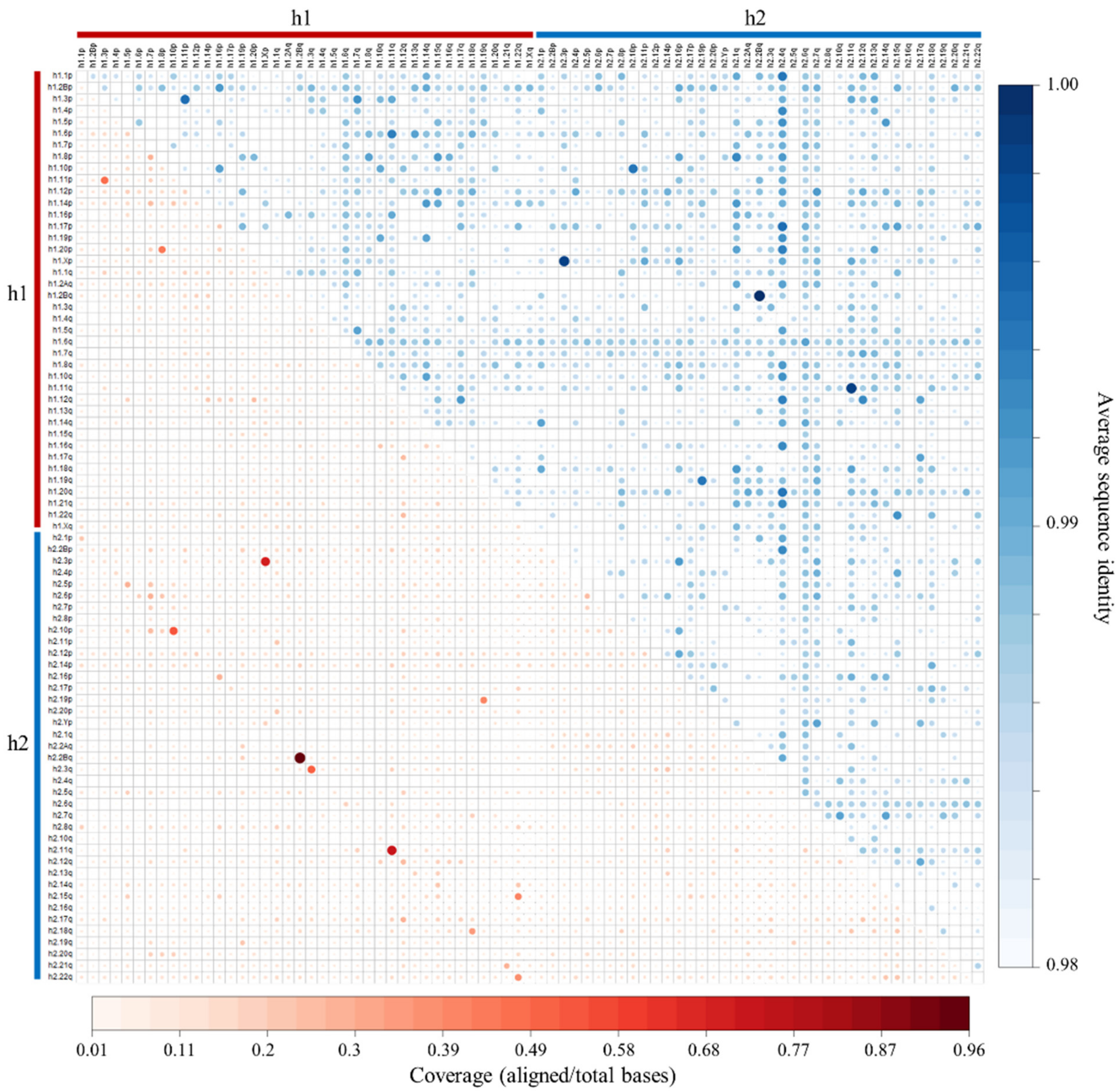

Supplemental Figure S10. Pairwise subterminal cap alignment in gorilla. Rows and columns show each of the subterminal caps present in the genome. The intensity of blue in the upper triangle shows the sequence average identity of pairwise alignment between subterminal caps of the respective row and column while the intensity of red in the lower triangle shows alignment coverage.

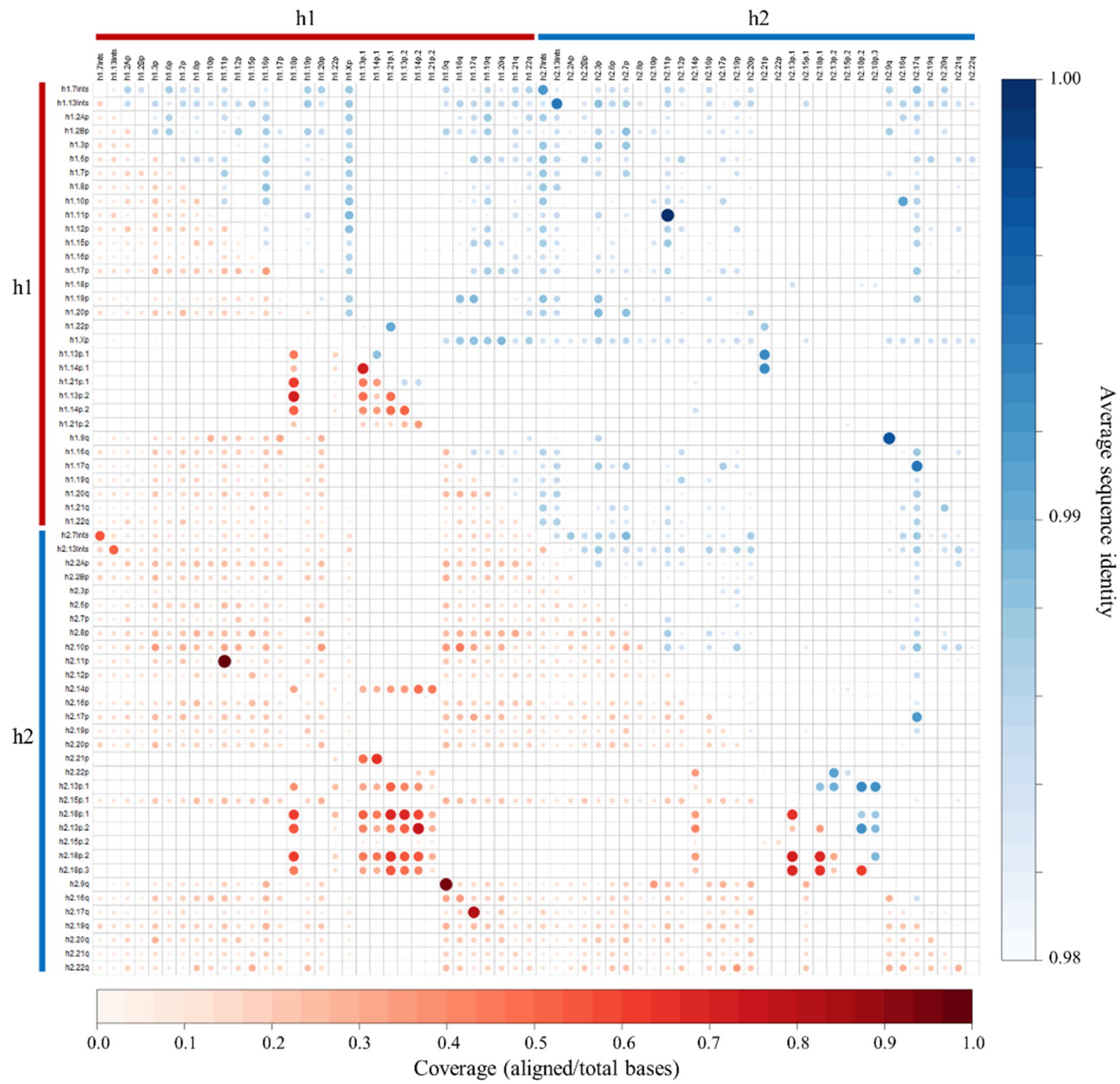

Supplemental Figure S11. Pairwise subterminal cap alignment in chimpanzee. Rows and columns show each of the subterminal caps present in the genome. The intensity of blue in the upper triangle shows the sequence average identity of pairwise alignment between subterminal caps of the respective row and column while the intensity of red in the lower triangle shows alignment coverage.

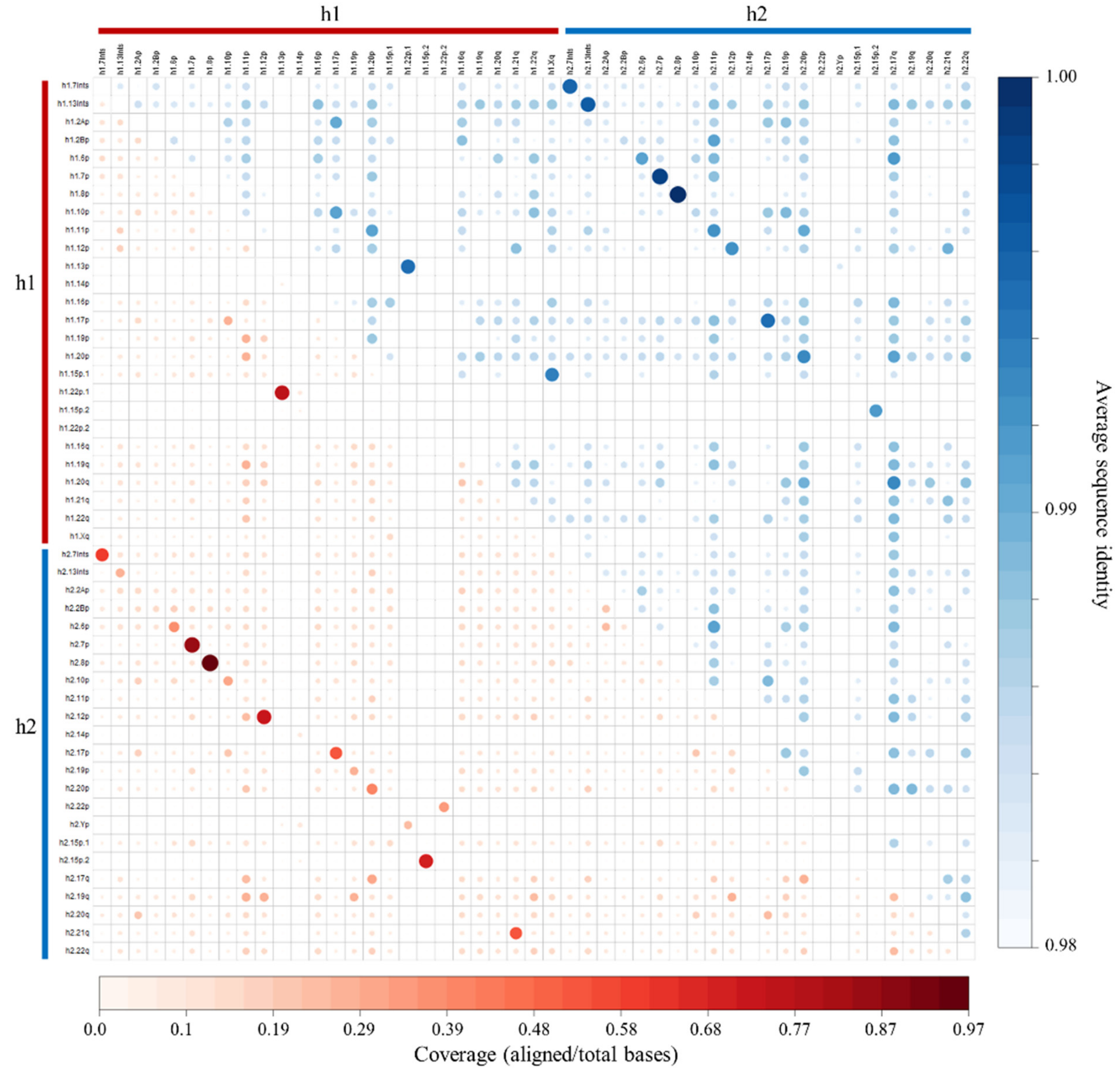

Supplemental Figure S12. Pairwise subterminal cap alignment in bonobo. Rows and columns show each of the subterminal caps present in the genome. The intensity of blue in the upper triangle shows the sequence average identity of pairwise alignment between subterminal caps of the respective row and column while the intensity of red in the lower triangle shows alignment coverage.

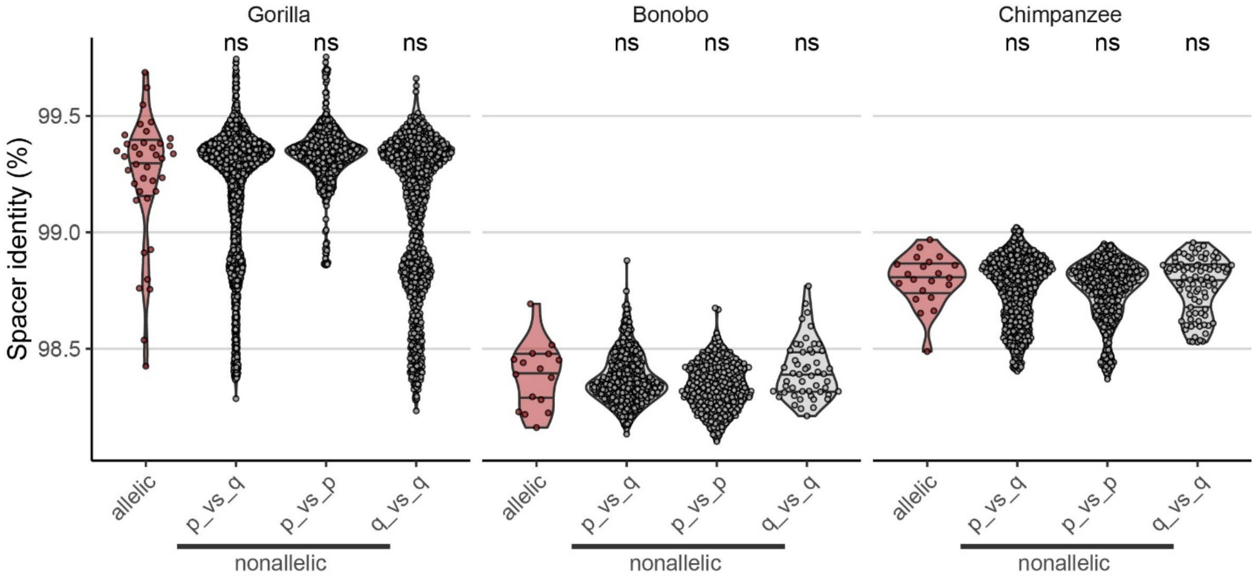

Supplemental Figure S13. Distribution of median sequence identity between SD spacers of allelic subterminal caps compared to nonallelic subterminal caps. Each dot represents the pairwise comparison. Two-sided permutation test significance is indicated on top, all of which were nonsignificant, $p<0.05$.

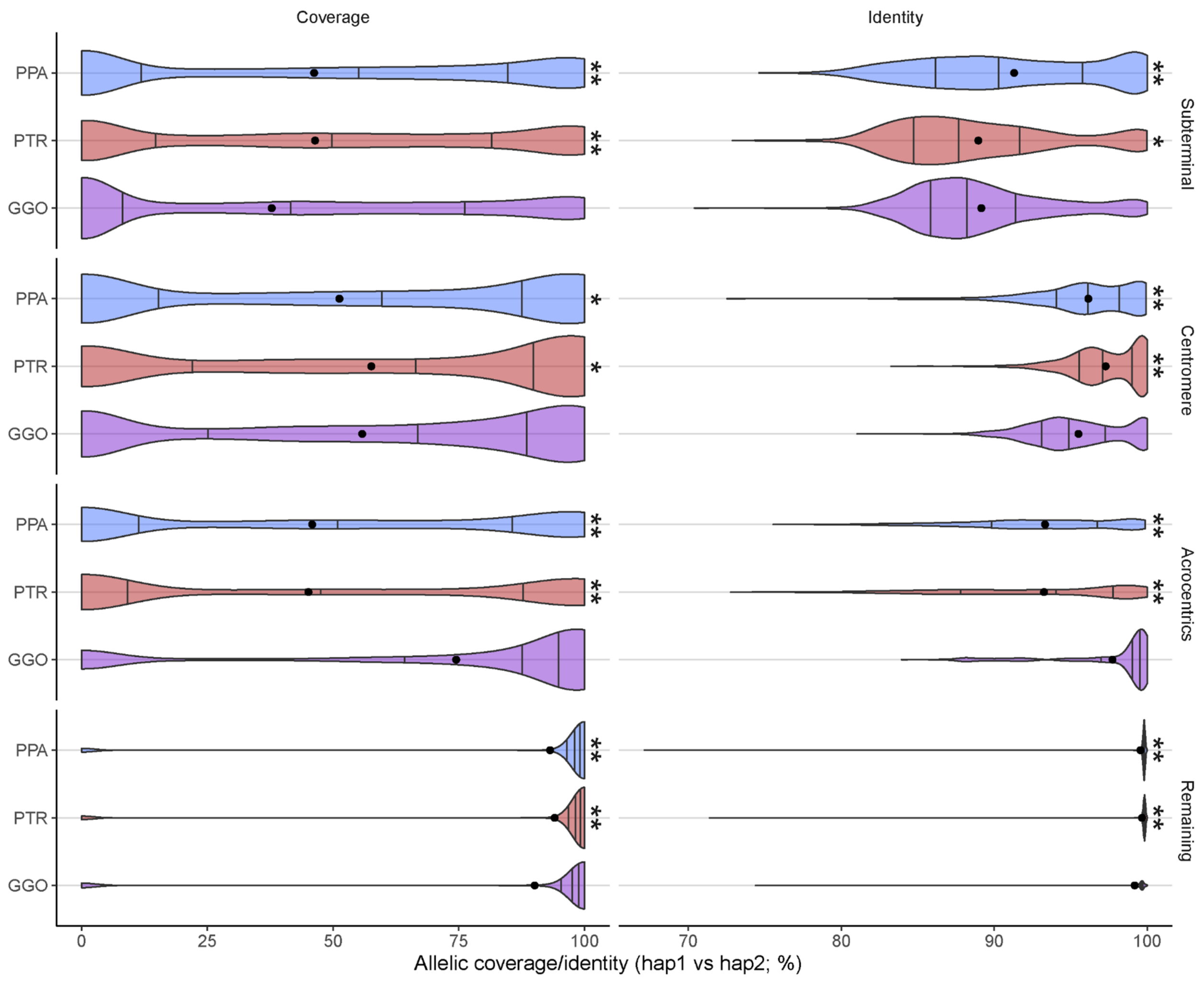

Supplemental Figure S14. Distribution of breadth of coverage and identity of 50 kbp nonoverlapping bins between haplotype 1 and haplotype 2, across subterminal heterochromatin, centromeres, acrocentric, and the remaining regions. The three vertical lines in each violin plot indicate first, second, and third quartiles, and the dot in each represents the mean. The asterisks indicate statistically significant differences of chimpanzee (PTR) and bonobo (PPA) compared to gorilla (GGO), based on two-sided Wilcox test; * and ** indicate $p<0.05$ and $p<0.0001$, respectively.

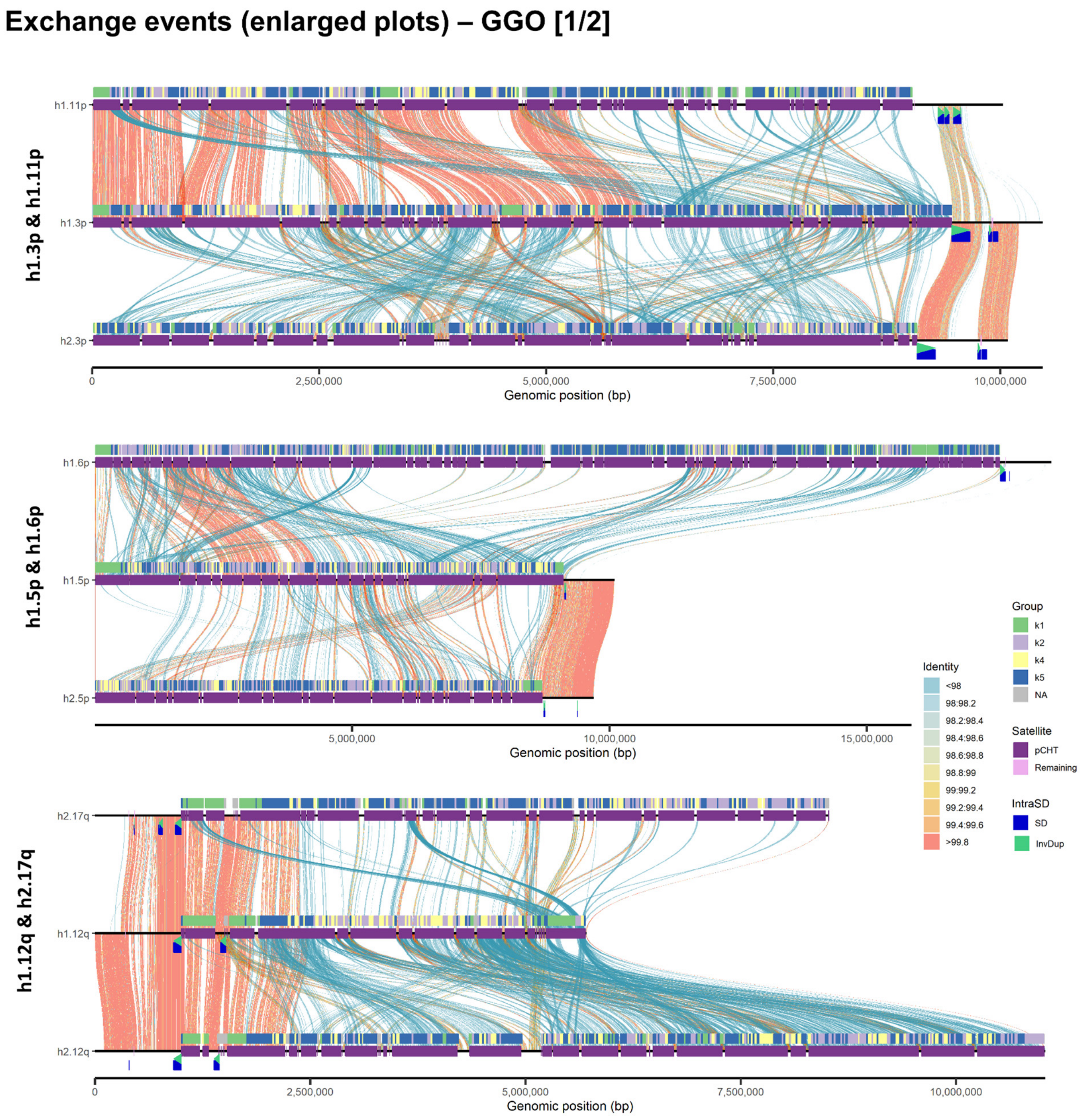

Supplemental Figure S15. Additional nonhomologous sequence exchanges of gorilla. Identity of alignment is scaled by blue to red. For each of the subterminal cap sequence, tracks from left to right indicate higher-order pCht blocks, satellites, and SDs. Putative exchange regions indicated by the long contiguous red alignment blocks between nonhomologous subterminal pairs.

Exchange events – GGO [2/2]

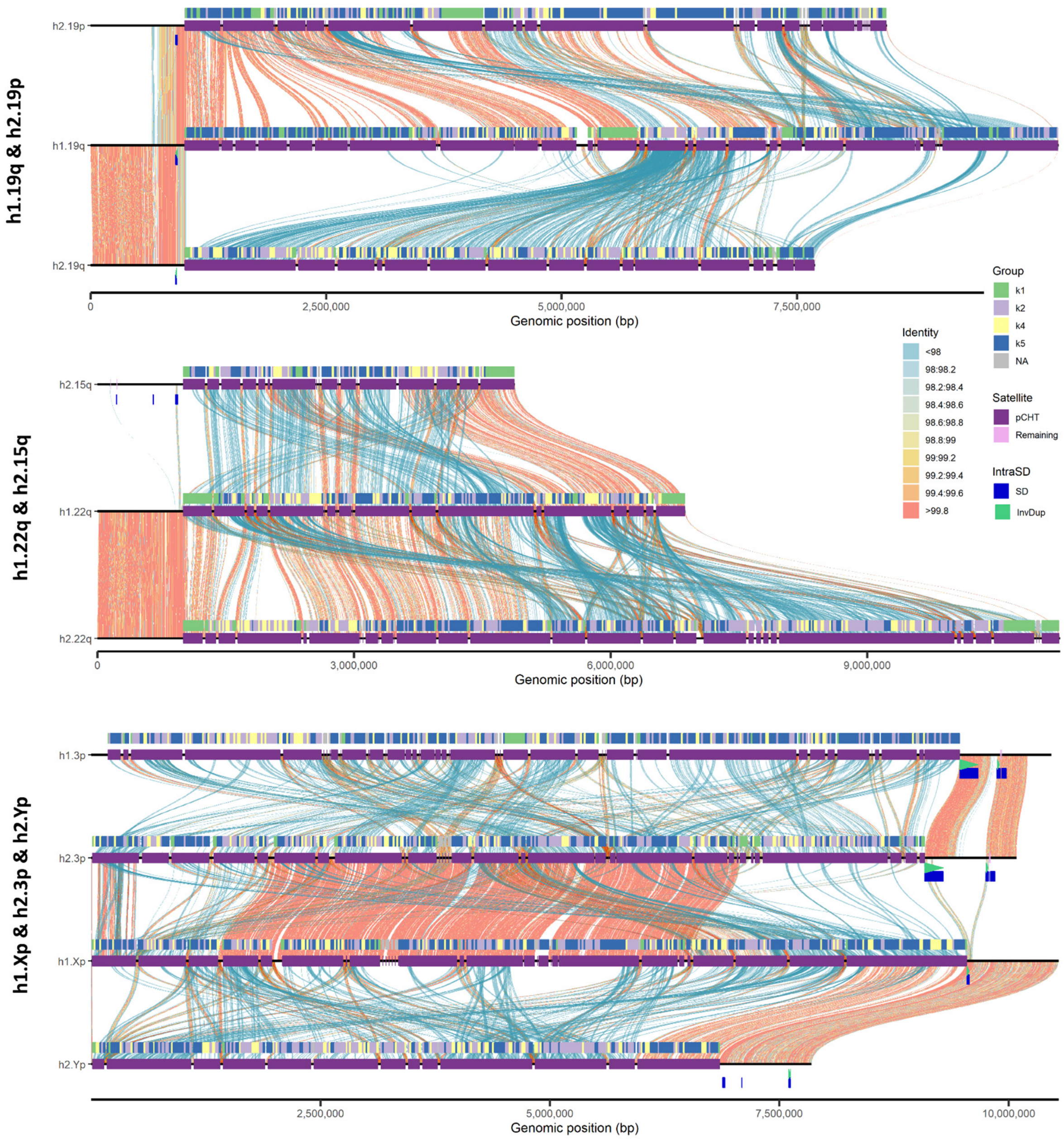

Supplemental Figure S16. Additional nonhomologous sequence exchanges of gorilla 2. Identity of alignment is scaled by blue to red. For each of the subterminal cap sequence, tracks from left to right indicate higher-order pCht blocks, satellites, and SDs. Putative exchange regions indicated by the long contiguous red alignment blocks between nonhomologous subterminal pairs.

Exchange events – PTR

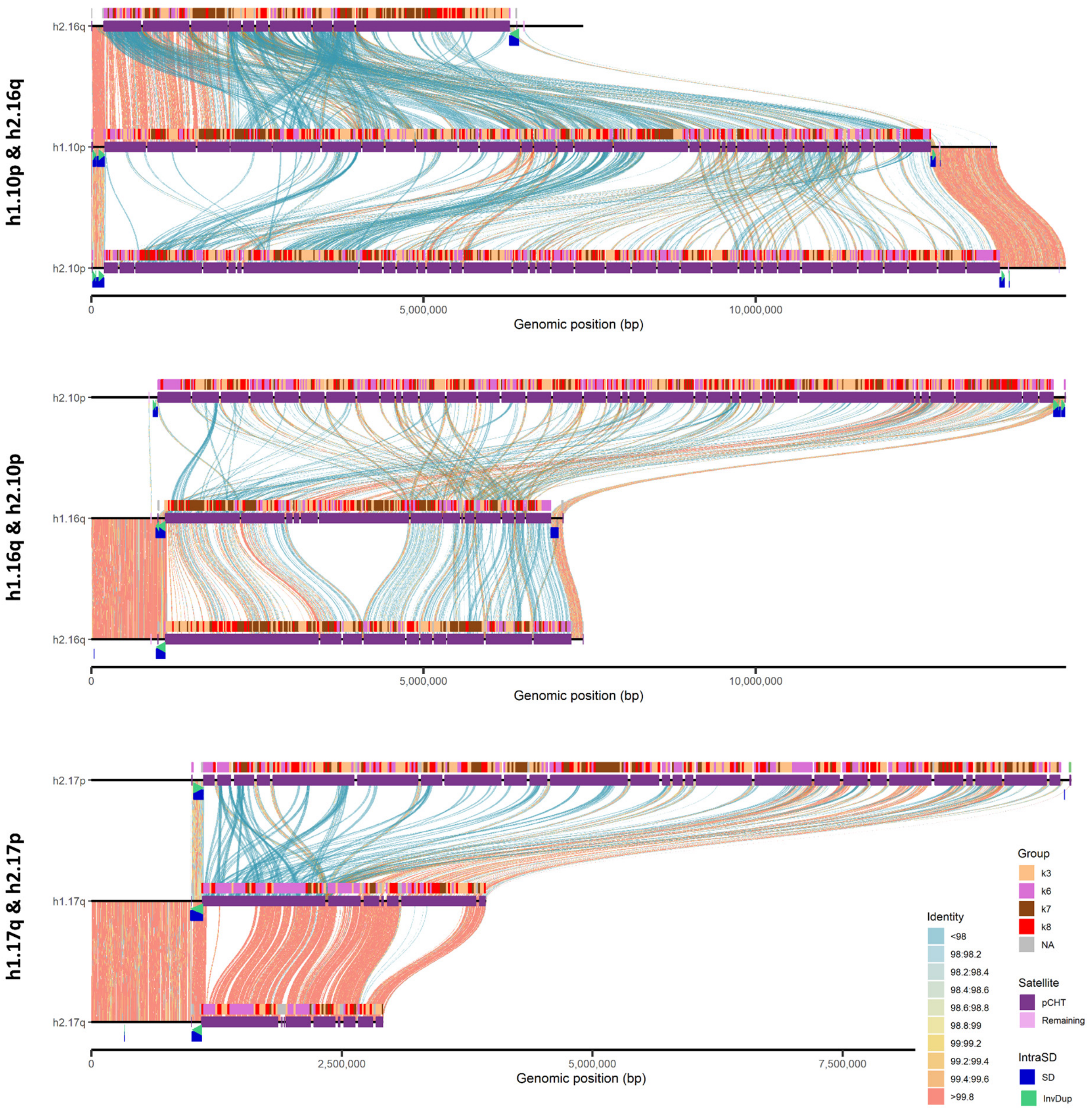

Supplemental Figure S17. Additional nonhomologous sequence exchanges of chimpanzee. Identity of alignment is scaled by blue to red. For each of the subterminal cap sequence, tracks from left to right indicate higher-order pCht blocks, satellites, and SDs. Putative exchange regions indicated by the long contiguous red alignment blocks between nonhomologous subterminal pairs.

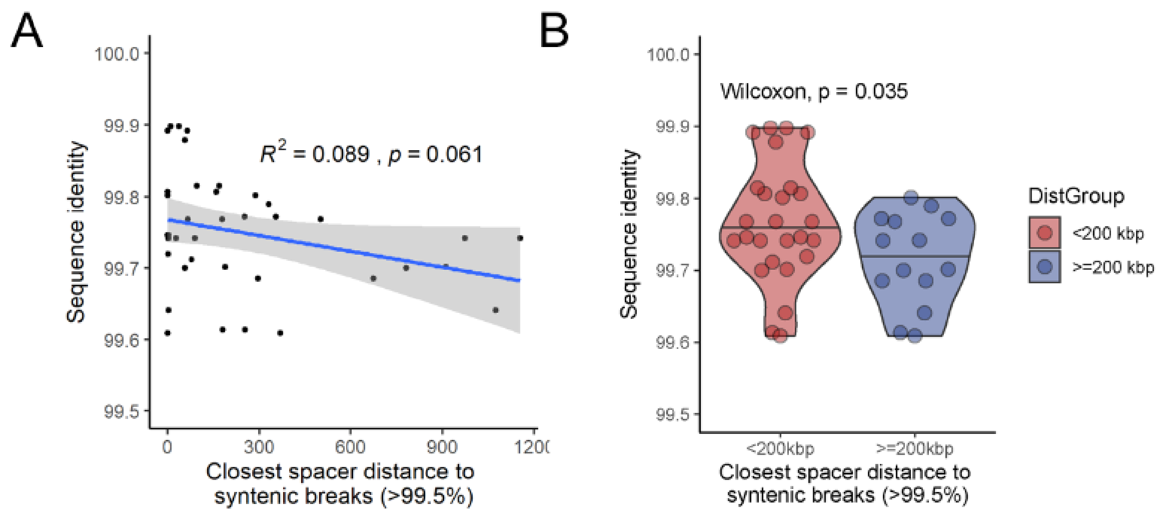

Supplemental Figure S18. SD Spacers distributed close to syntenic breaks between nonhomologous subterminal caps. (A) Correlation of sequence identity to closest spacer distance. The linear model is shown with a blue line with standard error indicated as the gray area. The correlation coefficient and *p*-value are indicated on the top. **(B)** Sequence identity distribution among putative subterminal sequence exchange events. The sequence identity tends to be significantly greater in the sequence exchange events, which contain the SD spacers in closer distances (<200 kbp) from the synteny breaks.

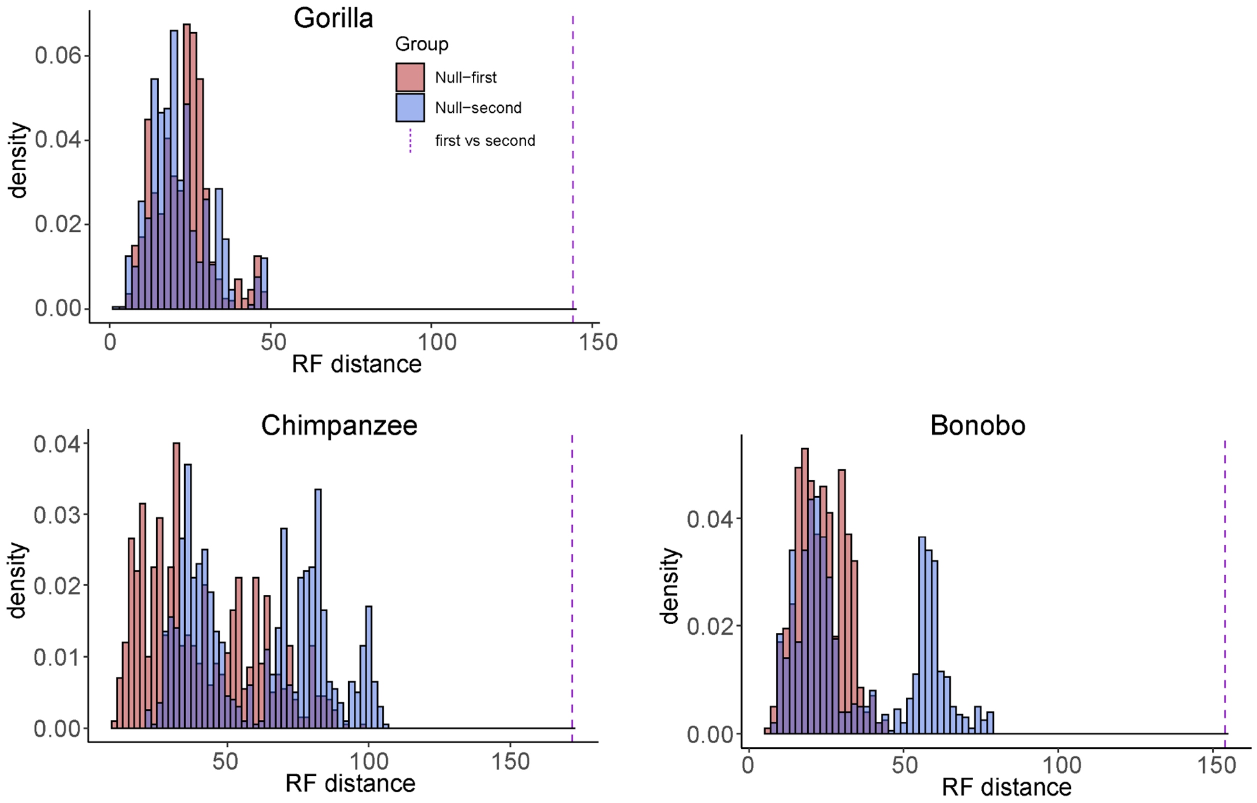

Supplemental Figure S19. Comparison of the topology of phylogeny from the first and second half of the SD spacers. Comparing the expected range of Robinson-Foulds distances among replicate trees (bootstrap replicate trees (n=1,000) to the consensus of the partial SD spacer trees), the difference between the two partial trees (purple dotted line) is significantly greater (p<0.001).

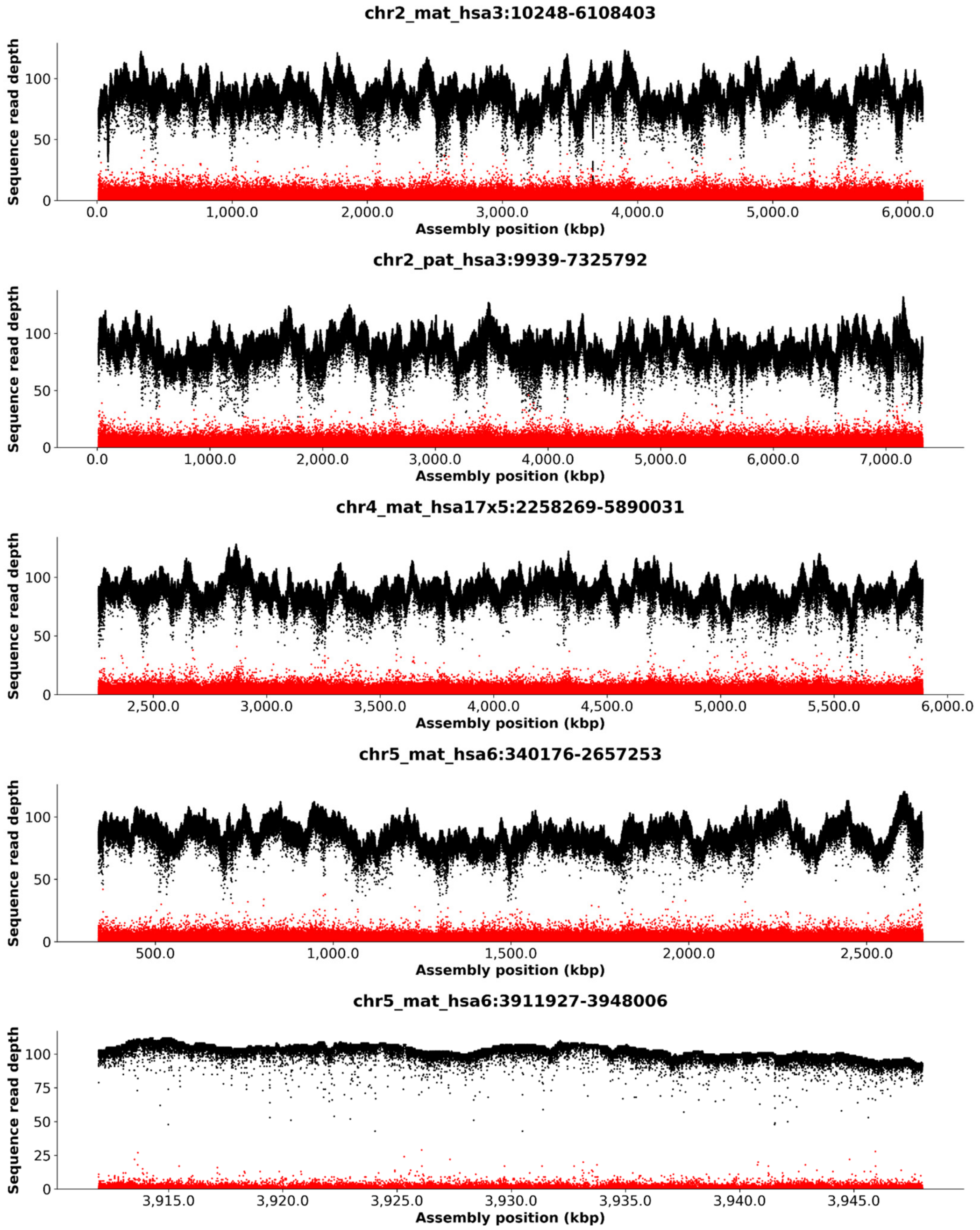

Supplemental Figure S20. Read-depth QC near the putative subterminal sequence exchange event in gorilla-1.

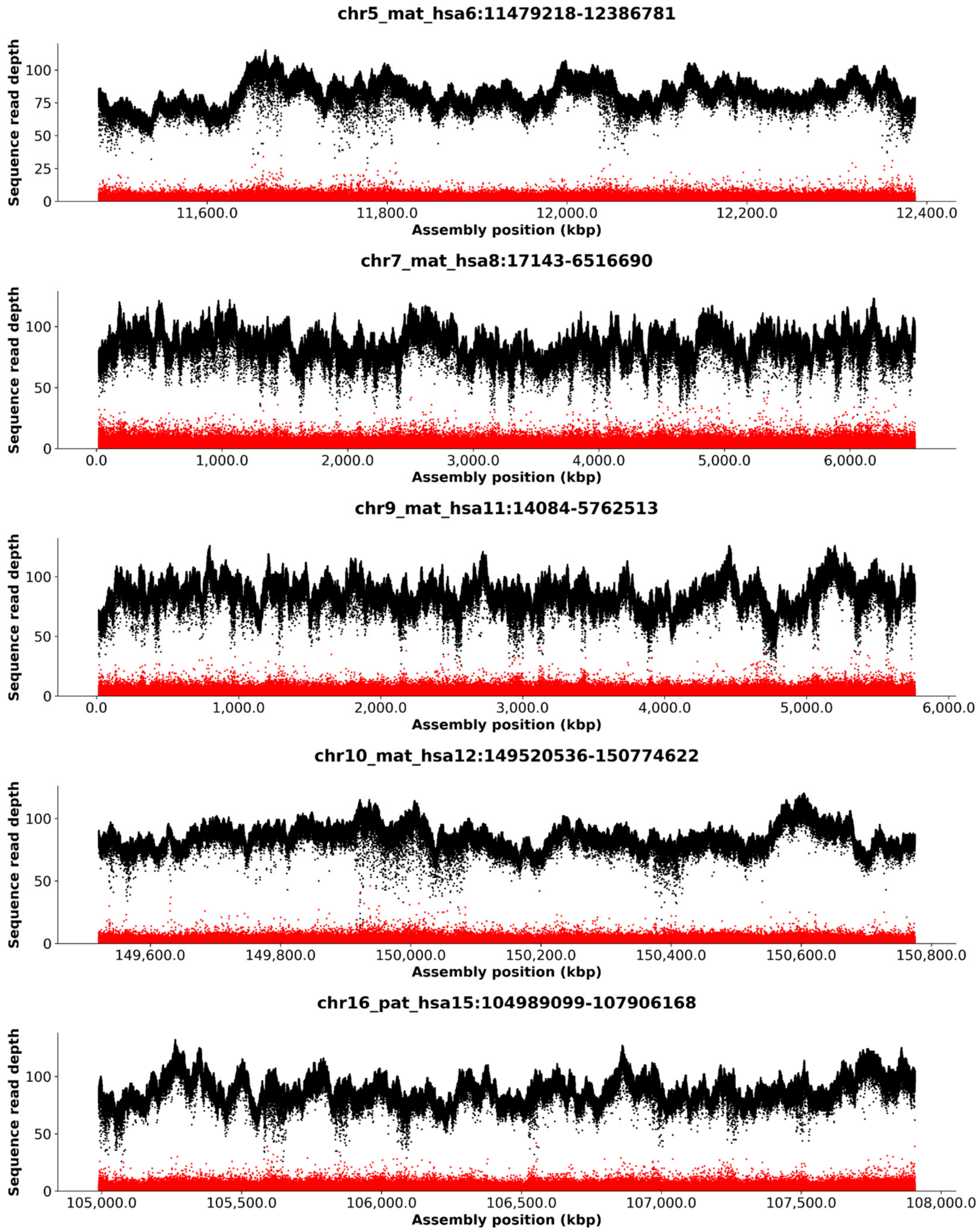

Supplemental Figure S21. Read-depth QC near the putative subterminal sequence exchange event in gorilla-2.

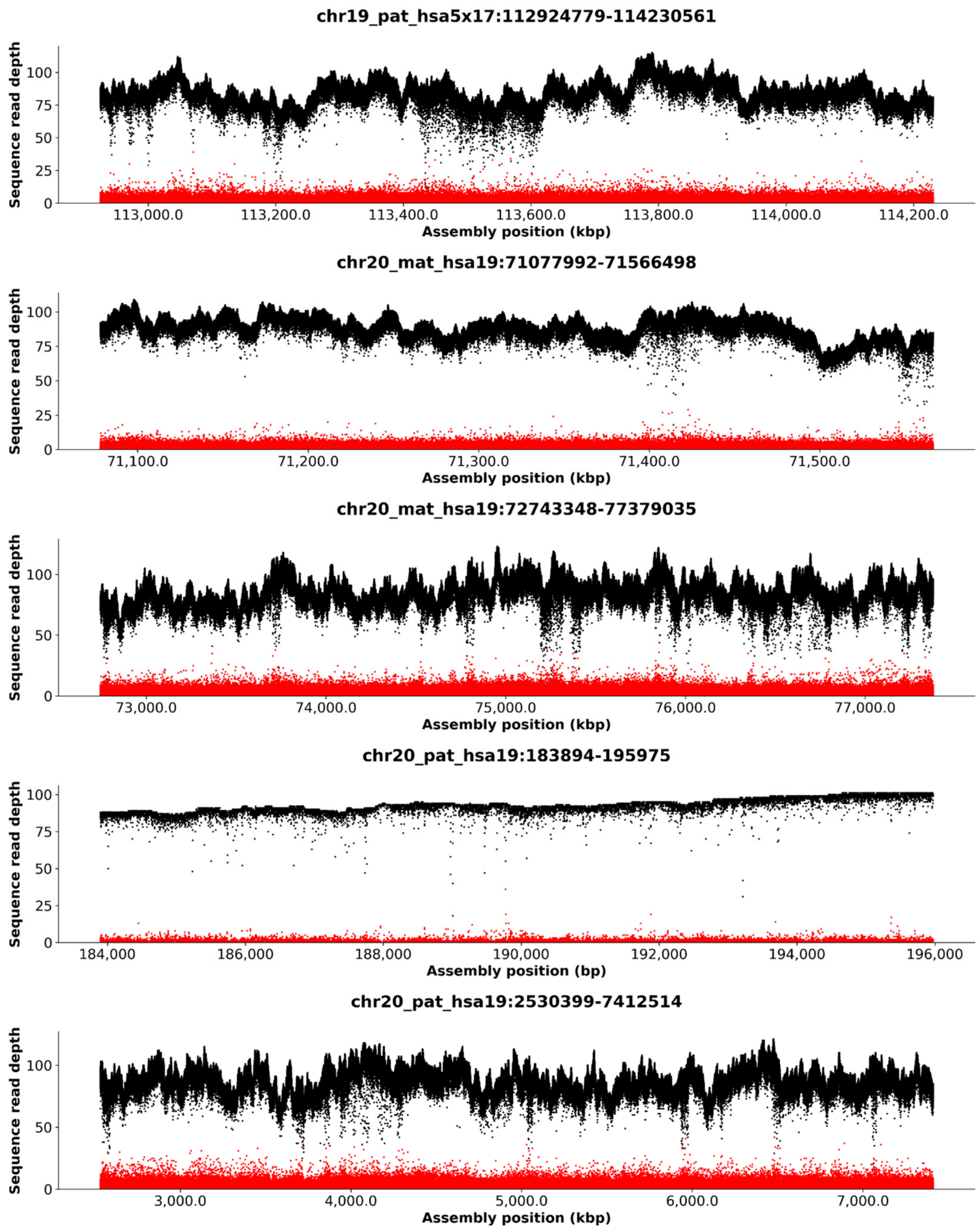

Supplemental Figure S22. Read-depth QC near the putative subterminal sequence exchange event in gorilla-3.

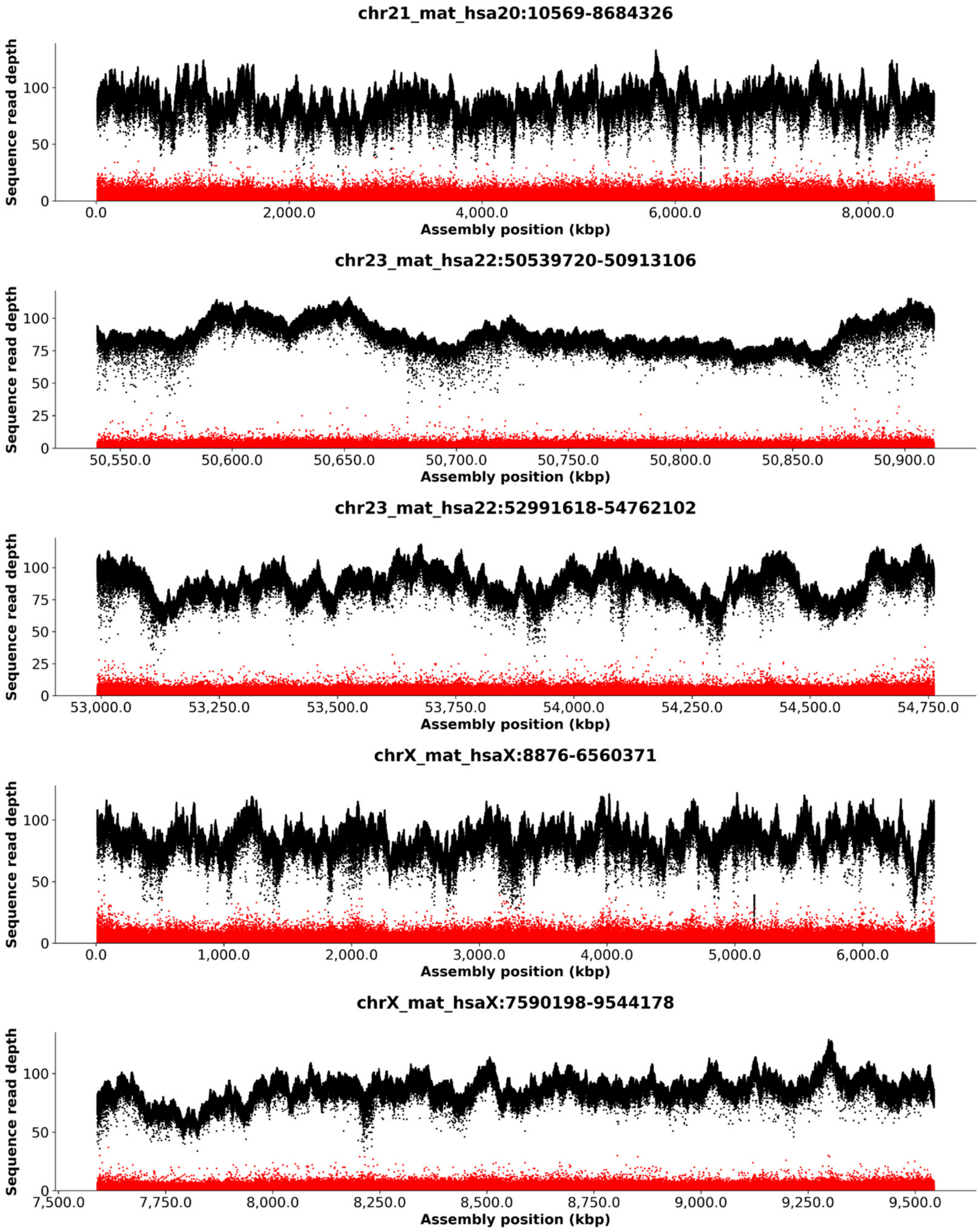

Supplemental Figure S23. Read-depth QC near the putative subterminal sequence exchange event in gorilla-4.

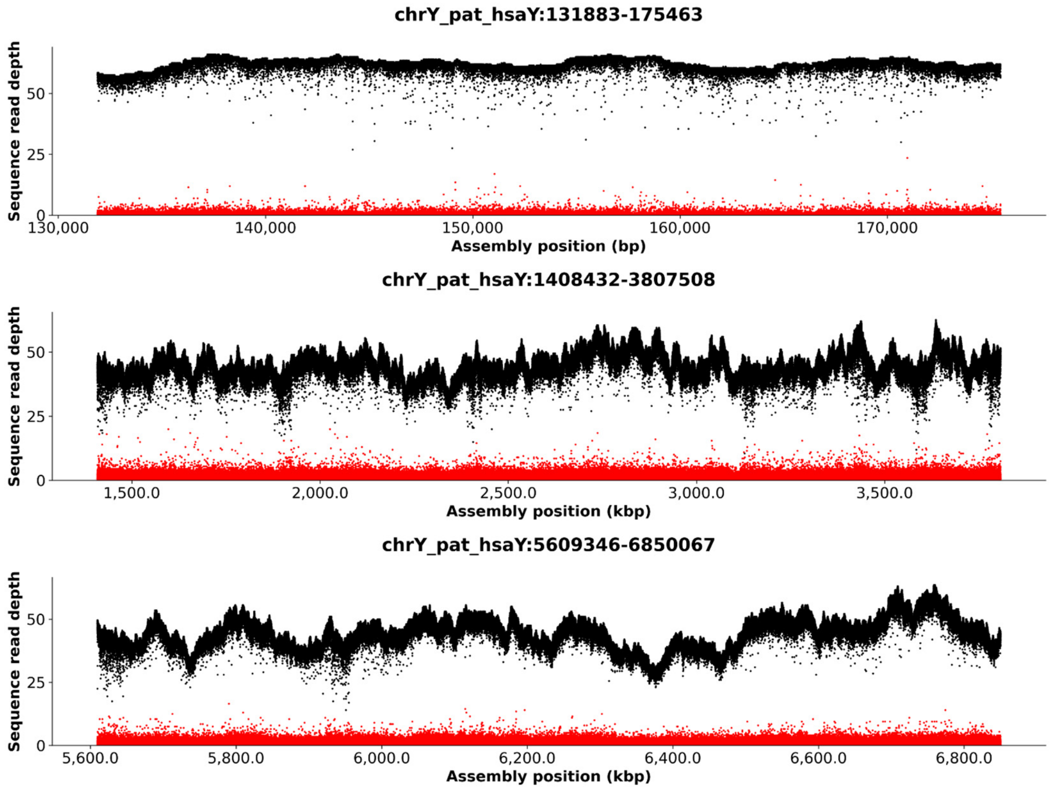

Supplemental Figure S24. Read-depth QC near the putative subterminal sequence exchange event in gorilla-5.

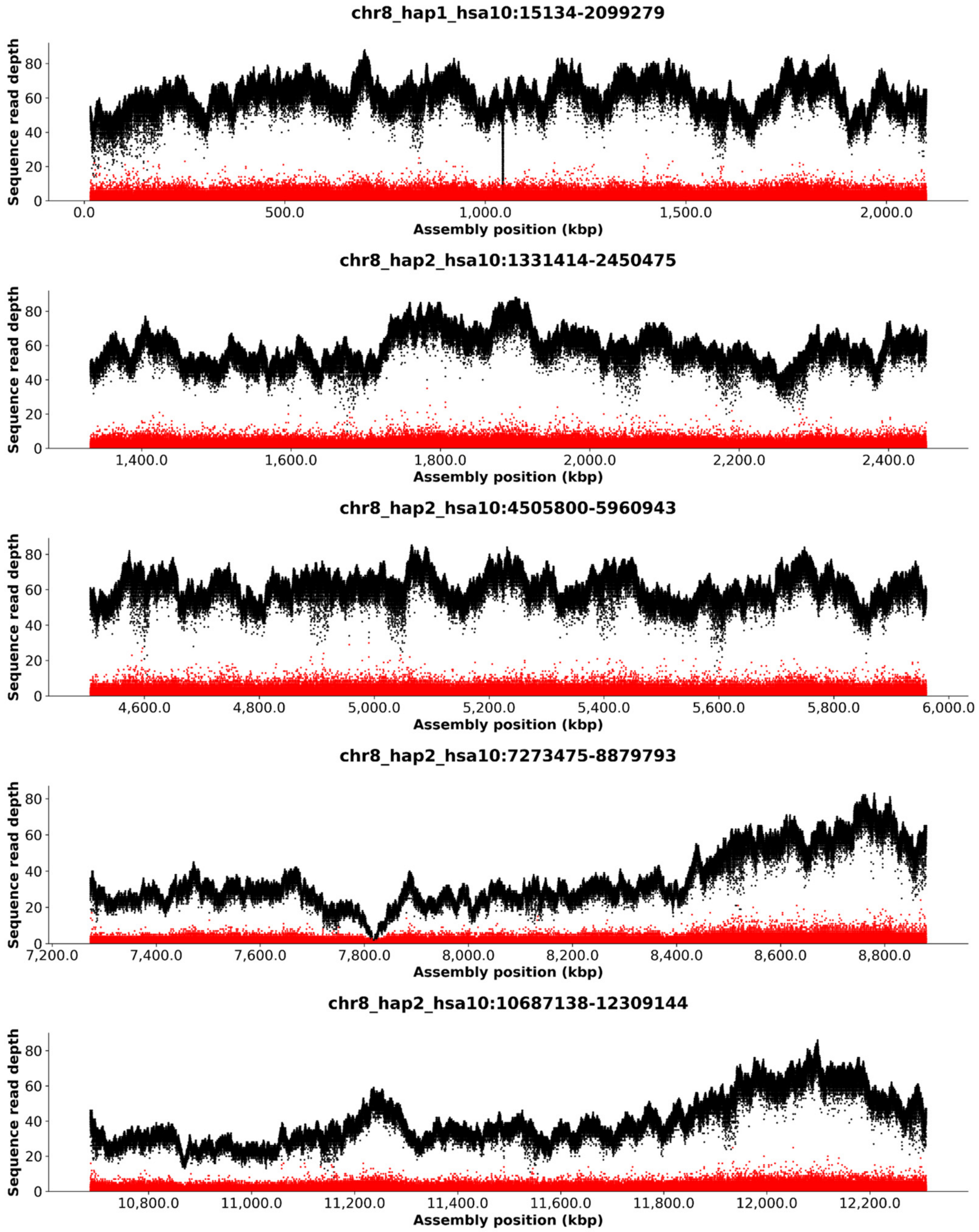

Supplemental Figure S25. Read-depth QC near the putative subterminal sequence exchange event in chimpanzee-1.

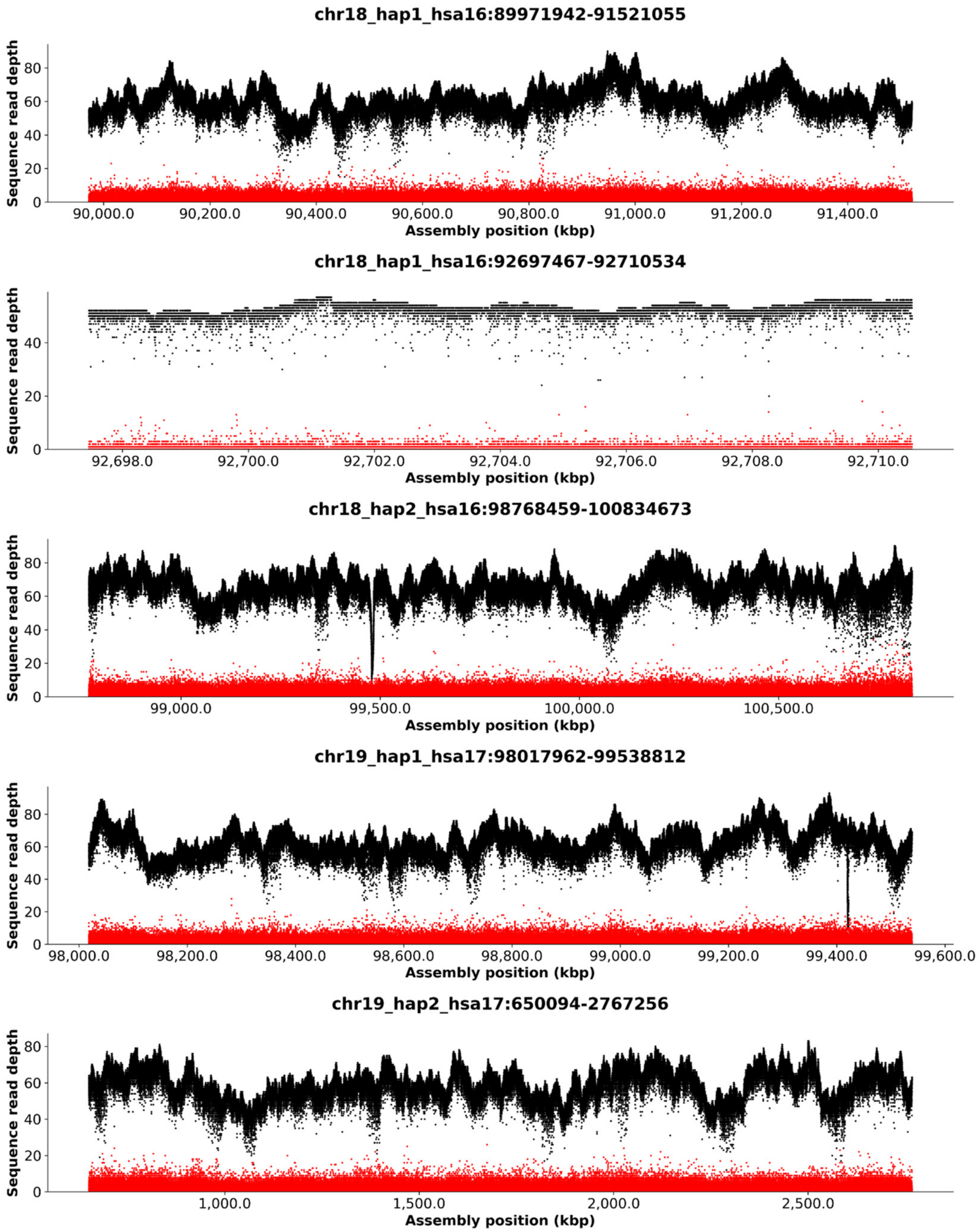

Supplemental Figure S26. Read-depth QC near the putative subterminal sequence exchange event in chimpanzee-2.

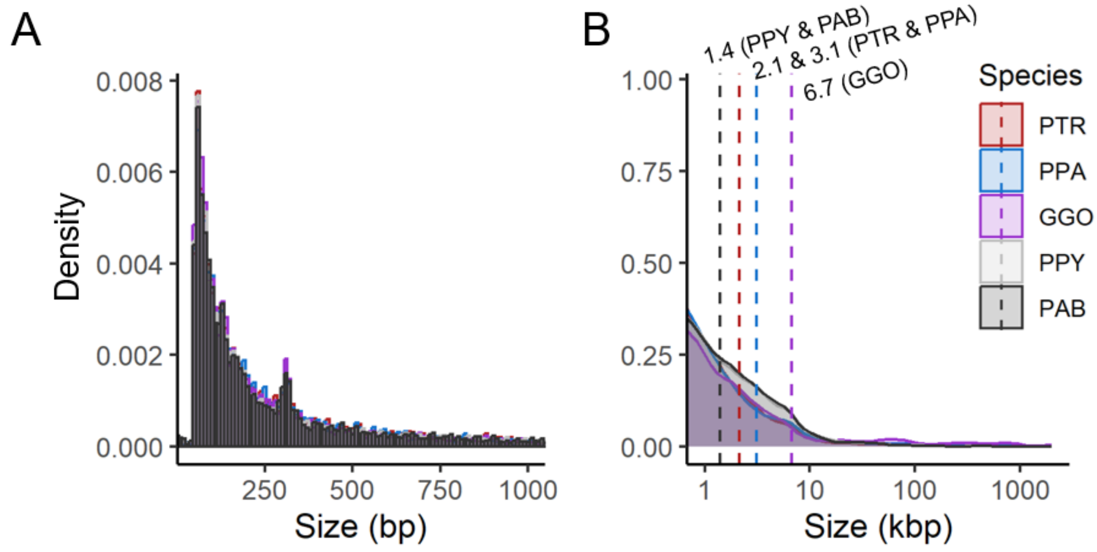

Supplemental Figure S27. Size distribution of nonhuman ape insertions with respect to human genome. (A) Size distribution of relatively smaller, <1 kbp insertions. **(B)** Size distribution of >1 kbp insertions. The dotted lines indicate the mean.

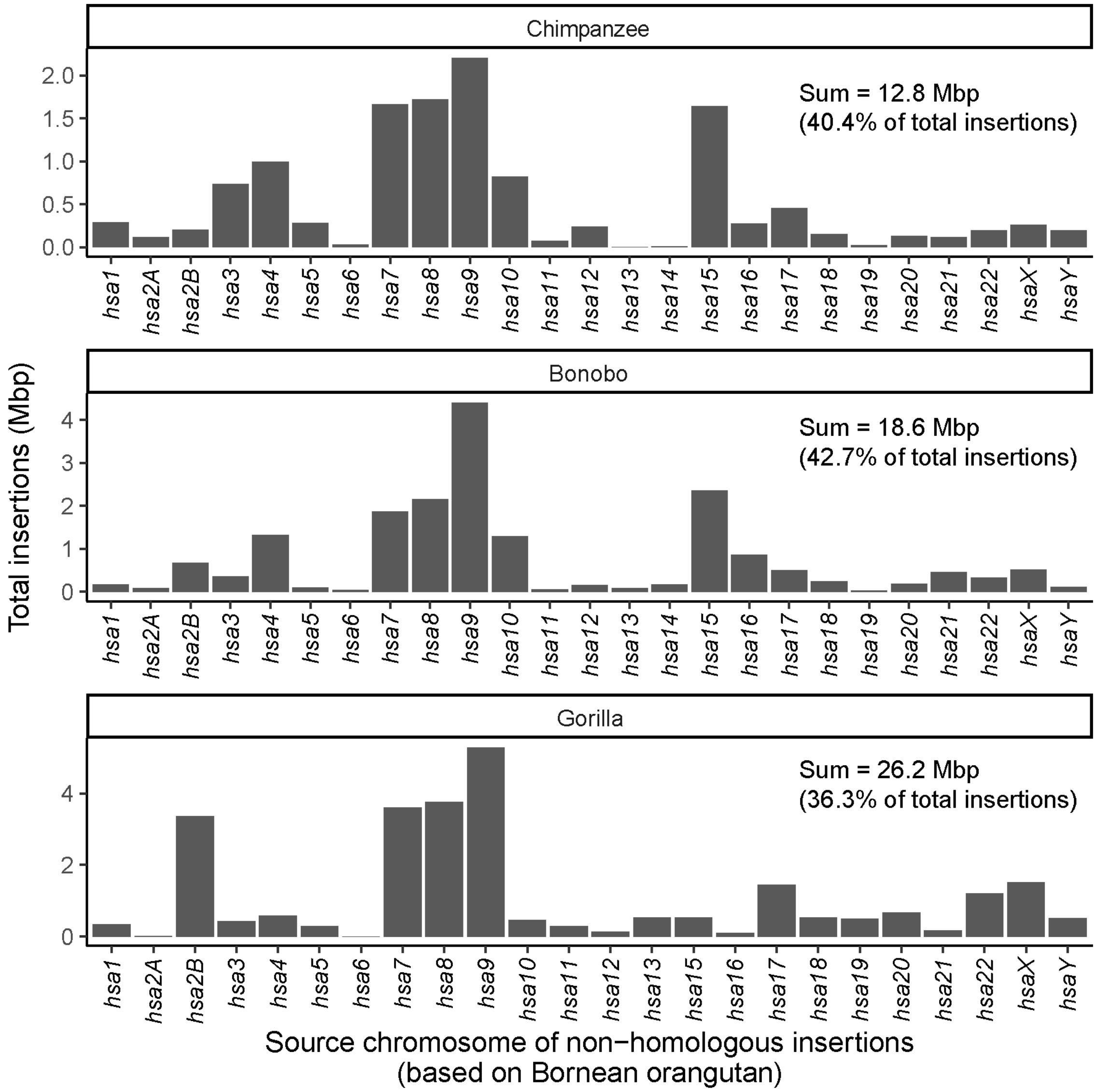

Supplemental Figure S28. Quantification of source of nonhomologous insertions identified in African great ape subterminal boundaries (2 Mbp upstream). The source chromosome was determined by alignment to the primary haplotype of Bornean orangutan genome.

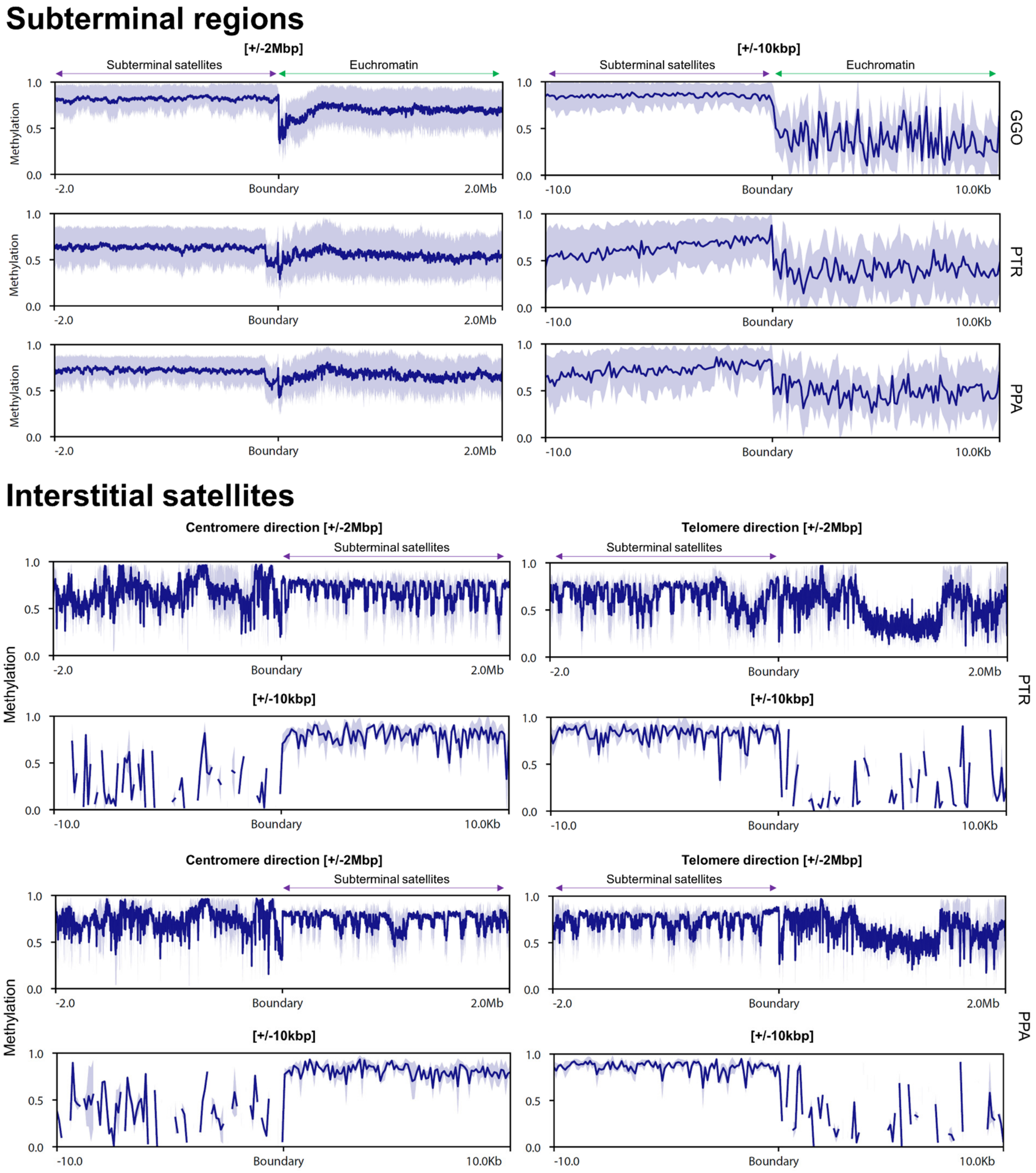

Supplemental Figure S29. Methylation at the boundaries of subterminal caps (top) or interstitial pCht satellites (bottom) in gorilla (GGO), chimpanzee (PTR) and bonobo (PPA).

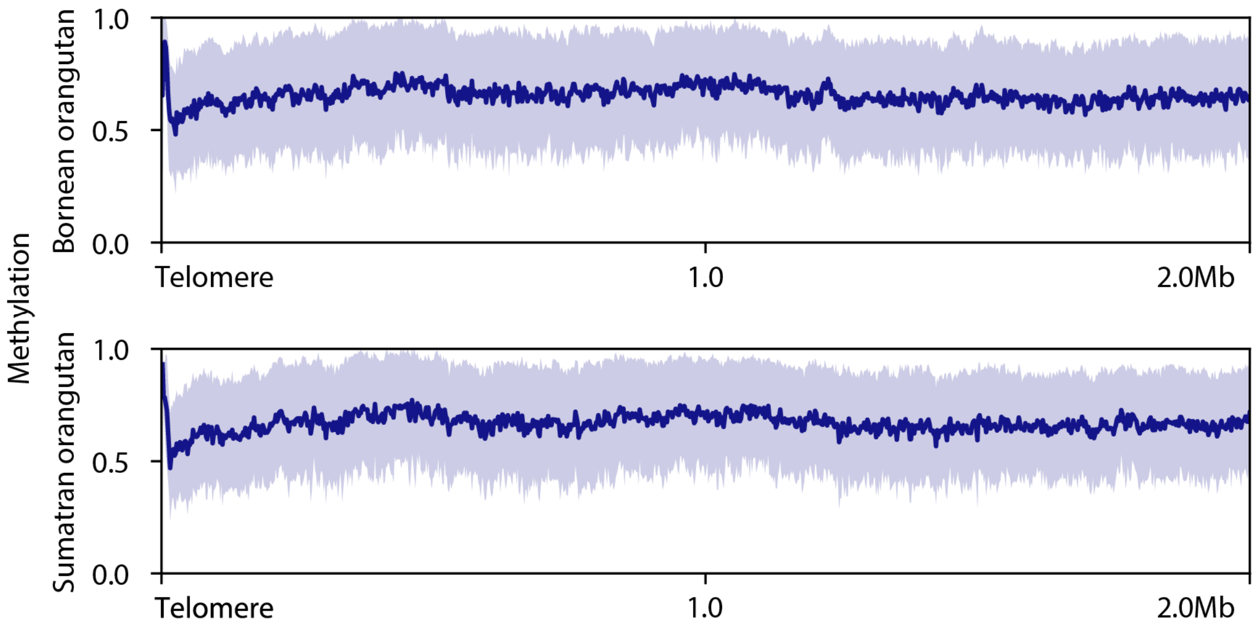

Supplemental Figure S30. Methylation across the 2 Mbp downstream of telomeres in Bornean and Sumatran orangutans.

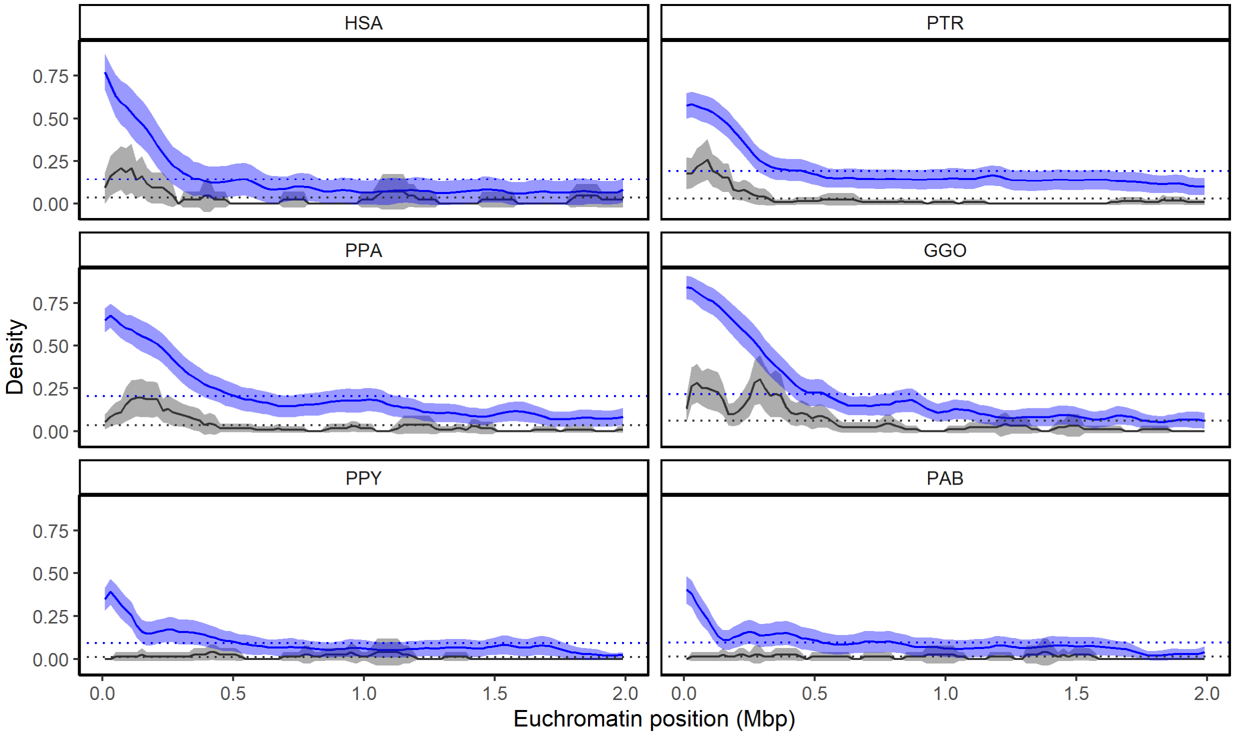

Supplemental Figure S31. Density of SDs and novel genes at the 2 Mbp upstream of the tip of the chromosomes or subterminal satellites. The transparent area indicates 95% interval of the observed density. Dotted horizontal line indicates the mean density.

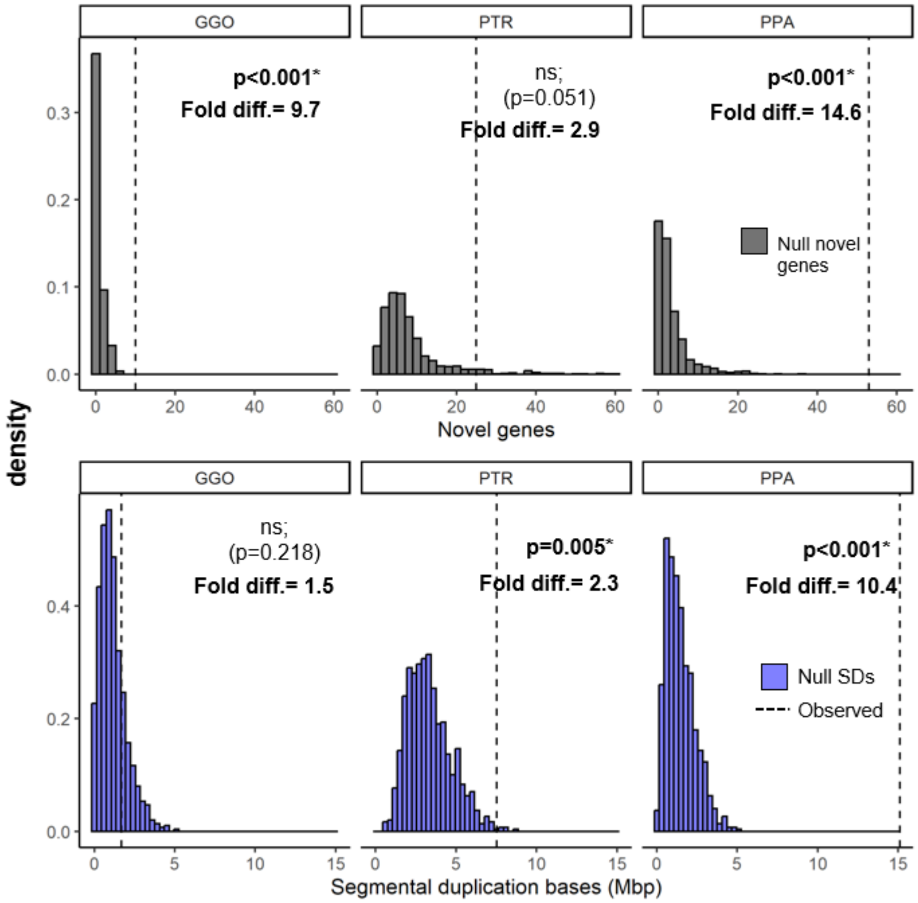

Supplemental Figure S32. Enrichment of SDs and lineage-specific genes at the non-StSat/acrocentric arms (2 Mbp upstream) of African great apes.

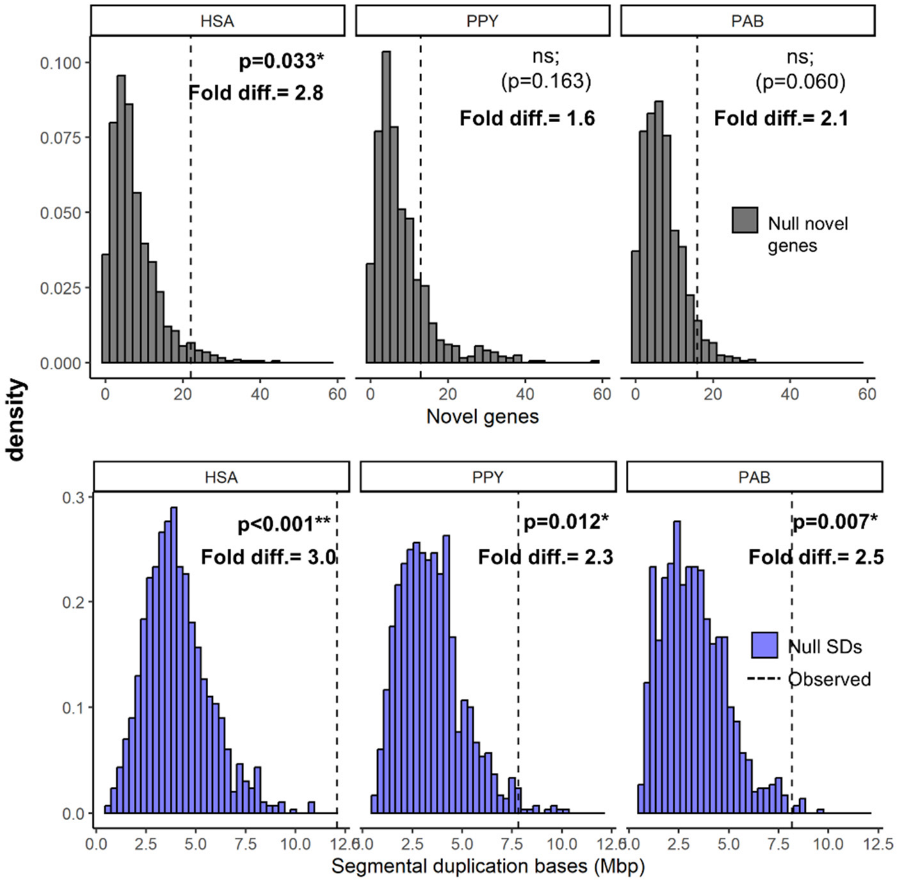

Supplemental Figure S33. Enrichment of SDs and lineage-specific genes at the 2 Mbp terminal regions of non-StSat/acrocentric arms.

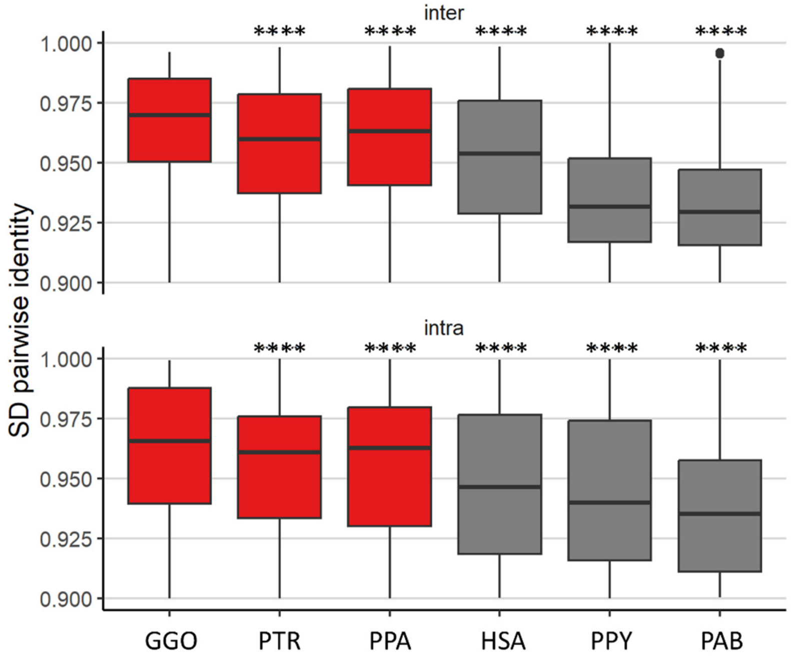

Supplemental Figure S34. Distribution of the pairwise identity of SDs located 2 Mbp upstream of chromosomal ends or subterminal satellites. The top panel shows the distribution for interchromosomal SDs, while the bottom panel shows intrachromosomal SDs. Two-sided Wilcox test compared to gorilla is indicated on the top of each box plot. **** indicates p<2.2e-16.

Supplemental data legend

Supplemental Data. Allelic alignments (available in a separate PDF file). Chromosome-by-chromosome comparison between allelic pairs of subterminal cap sequences. For each of the species (PTR: chimpanzee, PPA: bonobo, and GGO: gorilla), subterminal caps are displayed, aligning haplotype 2 (top) to haplotype 1 (bottom). In each comparison, the annotation tracks from the top indicate higher order block, followed by satellite tracks, inverted duplications (InvDup) in green, and lastly segmental duplications (SDs) at the bottom. Identity of alignment is indicated by blue to red.

Supplemental table legends S1-S7

Supplemental Table S1. Accessions of genomes. Available BioSample and BioProject IDs of respective species genomes used in this study.

Supplemental Table S2. Subterminal cap coordinates. Coordinates of subterminal cap including pCht satellites and spacer.

Supplemental Table S3. Spacer segmental duplication (SD) coordinates.

Supplemental Table S4. Iso-Seq read mapping statistics. Total number of processed Iso-Seq reads are summarized. Filter options are described in "Note" column.

Supplemental Table S5. Transcript data mapping to subterminal regions. Location of transcript models mapping in subterminal caps are summarized. Number of Iso-Seq reads supporting each transcript and the number of exons are indicated.

Supplemental Table S6. Novel gene and normalized read counts. Novel genes located within 2 Mbp subterminal boundary regions are summarized.

Supplemental Table S7. Gene ontology enrichment. Biological process, molecular function, and cellular component terms enriched by novel genes.
