# Supplementary material for: Epigenetic and evolutionary features of ape subterminal heterochromatin
Source: Genome Res. 2026 Jan;36(1):38–49. doi: 10.1101/gr.280987.125 (PMC12758386; doi:10.1101/gr.280987.125)
Supplement: Supplement 3 [file Supplemental_Data.zip › Supplemental_Data/README.rtf]

Supplemental Data legendSupplemental Data. Allelic alignments (available in a separate PDF file). Chromosome-by-chromosome comparison between allelic pairs of subterminal cap sequences. For each of the species (PTR: chimpanzee, PPA: bonobo, and GGO: gorilla), subterminal caps are displayed, aligning haplotype 2 (top) to haplotype 1 (bottom). In each comparison, the annotation tracks from the top indicate higher order block, followed by satellite tracks, inverted duplications (InvDup) in green, and lastly segmental duplications (SDs) at the bottom. Identity of alignment is indicated by blue to red.
