# Supplementary material for: Epigenetic and evolutionary features of ape subterminal heterochromatin
Source: Genome Res. 2026 Jan;36(1):38–49. doi: 10.1101/gr.280987.125 (PMC12758386; doi:10.1101/gr.280987.125)
Supplement: Supplement 3 [file Supplemental_Data.zip › Supplemental_Data/Supplemental_Data.pdf]

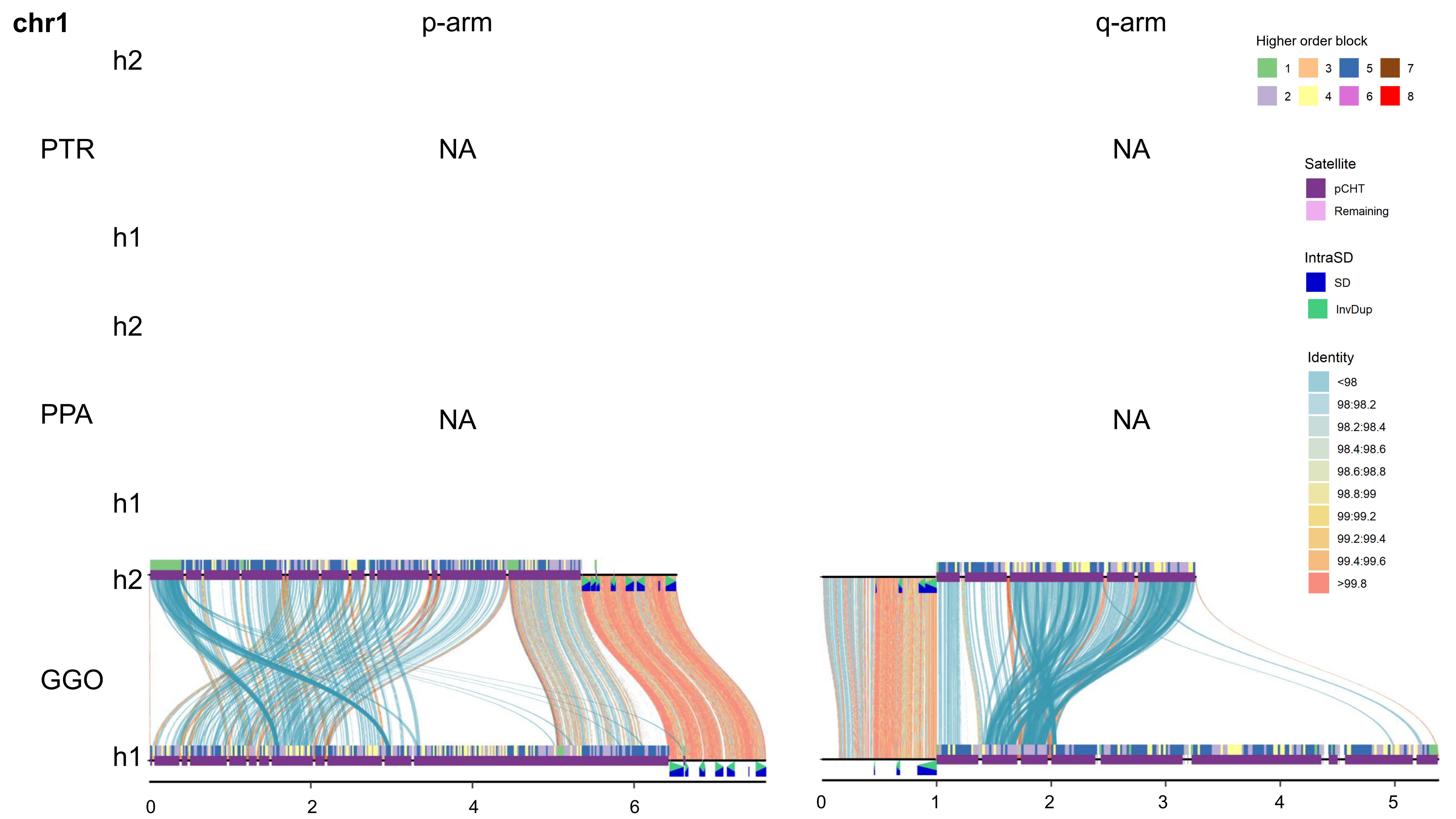
chr1
p-arm
q-arm
h2
PTR
NA
NA
h1
h2
PPA
NA
NA
h1
h2
GGO
h1
0
2
4
6
0
1
2
3
4
5
Higher order block
1
2
3
4
5
6
7
8
Satellite
pCHT
Remaining
IntraSD
SD
InvDup
Identity
<98
98:98.2
98.2:98.4
98.4:98.6
98.6:98.8
98.8:99
99:99.2
99.2:99.4
99.4:99.6
>99.8

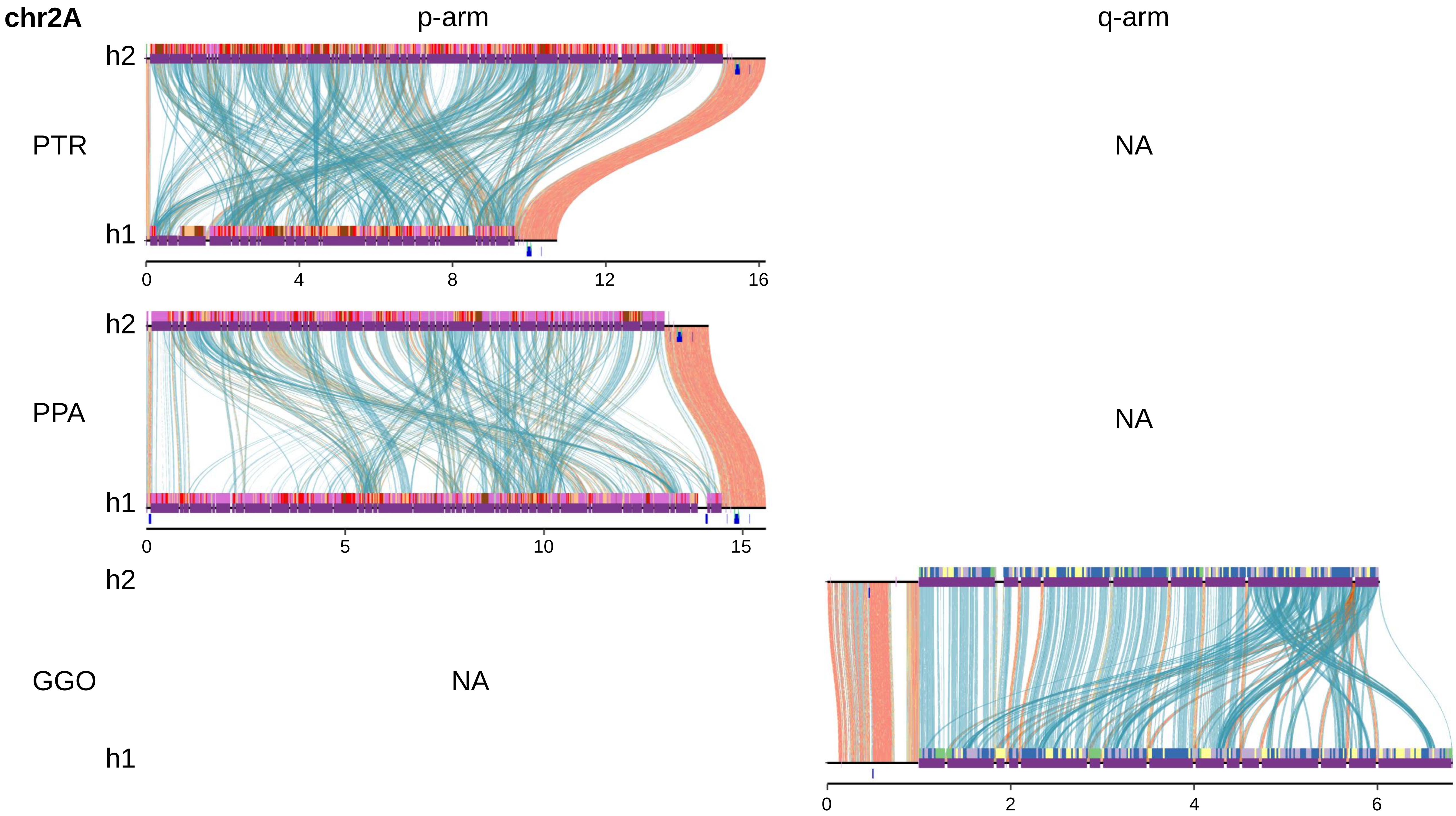

chr2A
p-arm
q-arm
h2
h1
PTR
NA
0
4
8
12
16
PPA
NA
0
5
10
15
GGO
NA
0
2
4
6

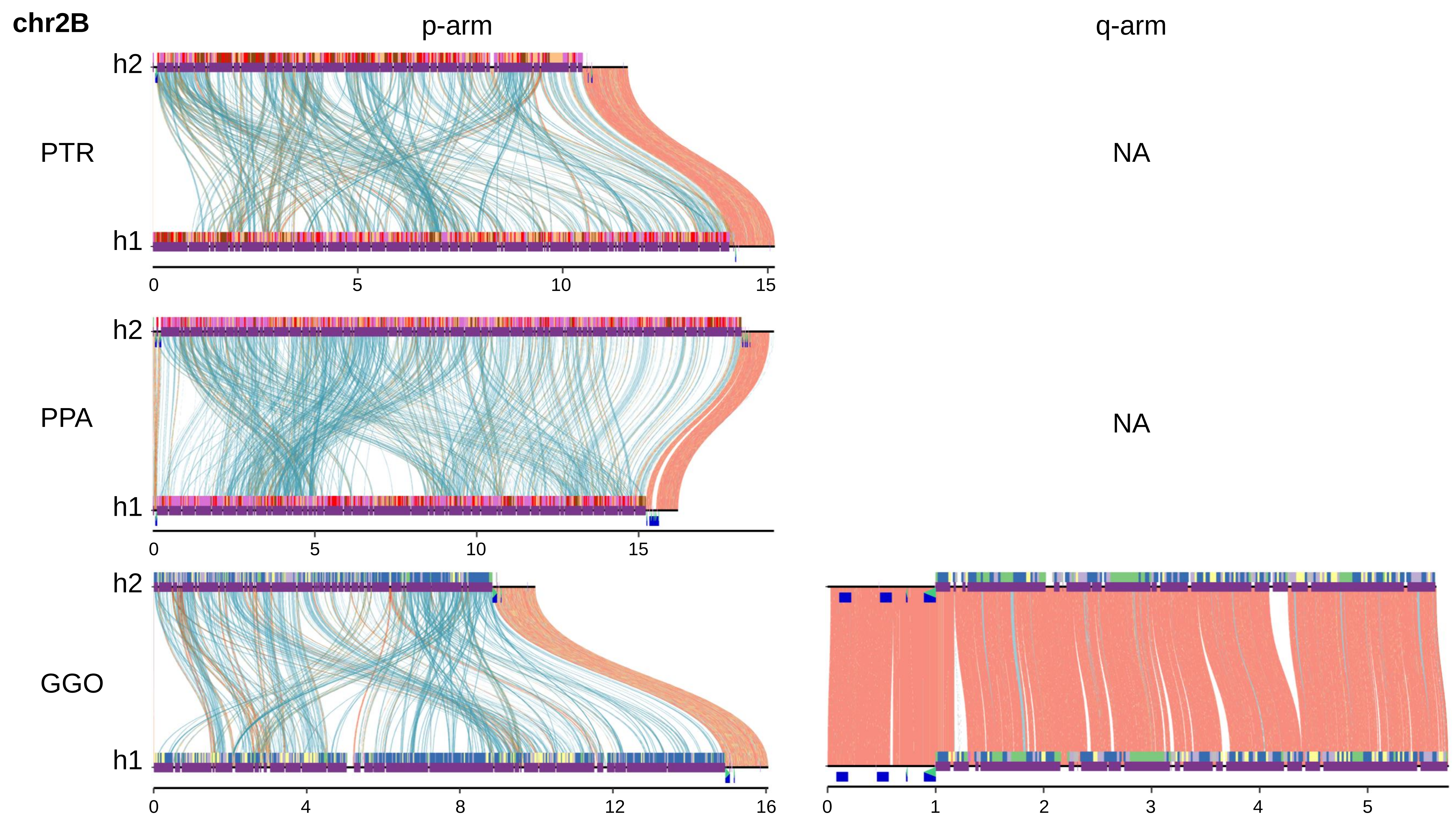
chr2B
p-arm
q-arm
h2
h1
PTR
NA
0
5
10
15
h2
h1
PPA
NA
0
5
10
15
h2
h1
GGO
0
4
8
12
16
0
1
2
3
4
5

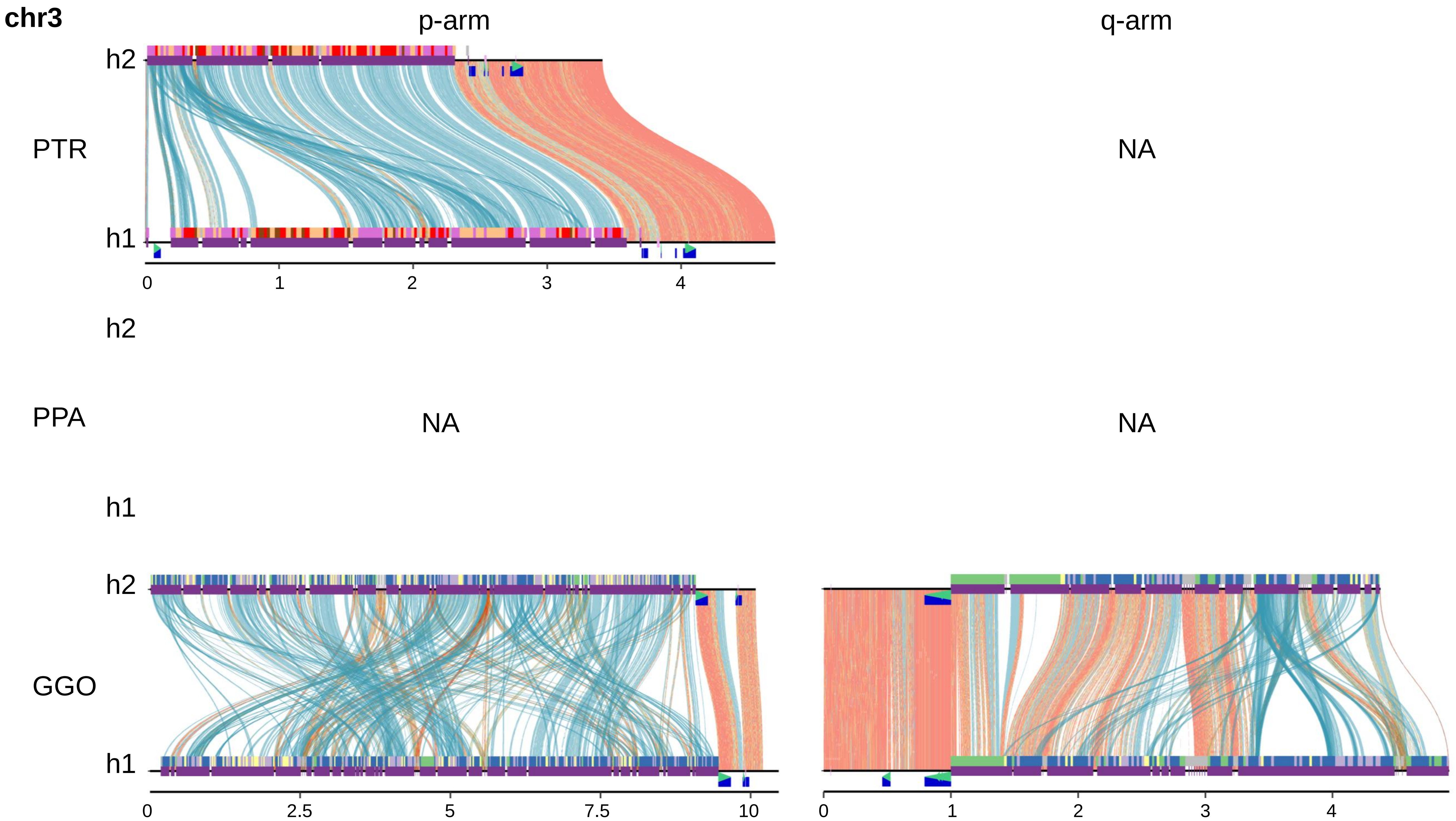
chr3
p-arm
q-arm
PTR
PPA
GGO
h2
h1
NA
NA
NA
0
1
2
3
4
2.5
5
7.5
10

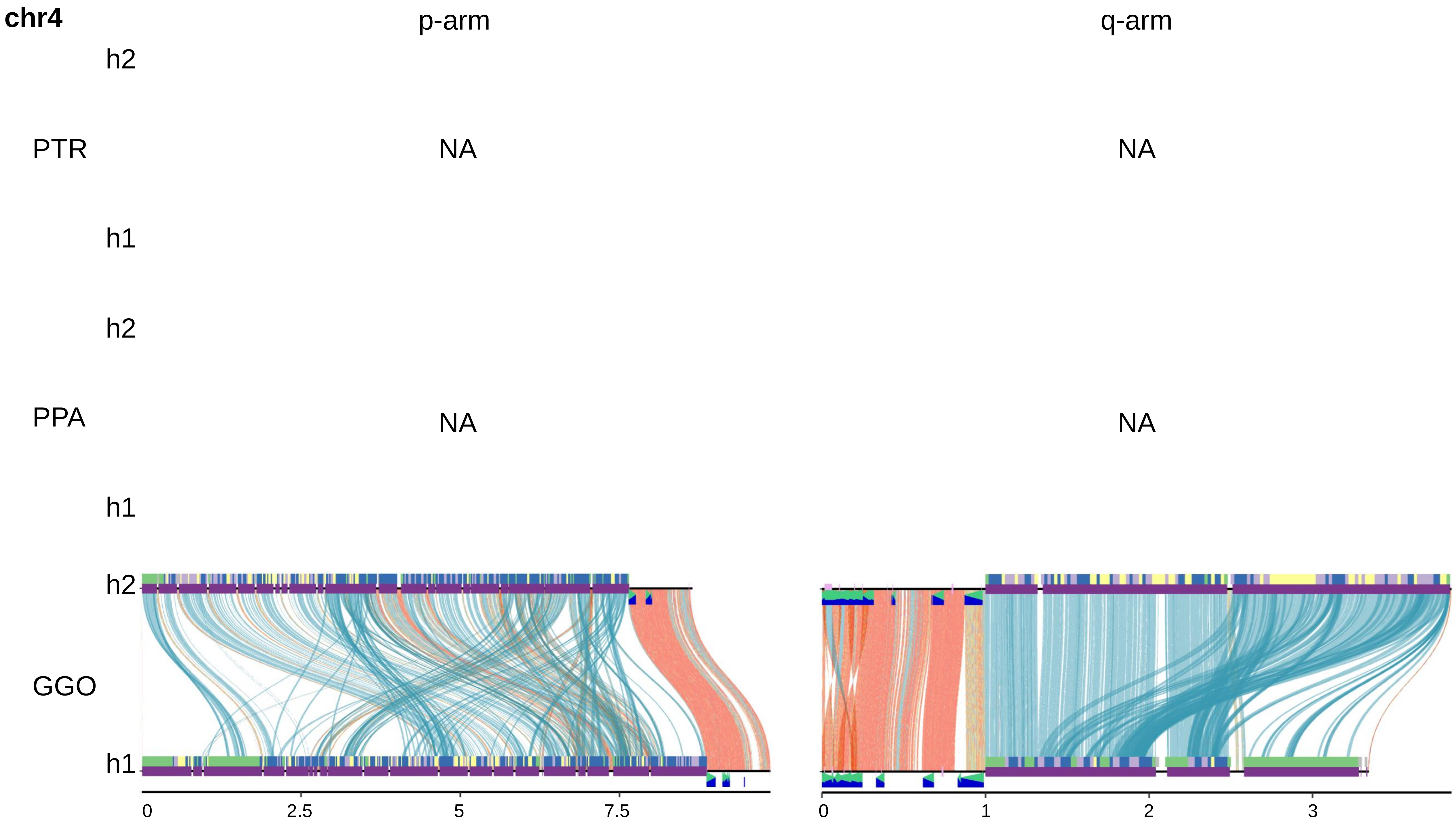
chr4
p-arm
q-arm
h2
PTR
NA
NA
h1
h2
PPA
NA
NA
h1
h2
GGO
h1
0
2.5
5
7.5
0
1
2
3

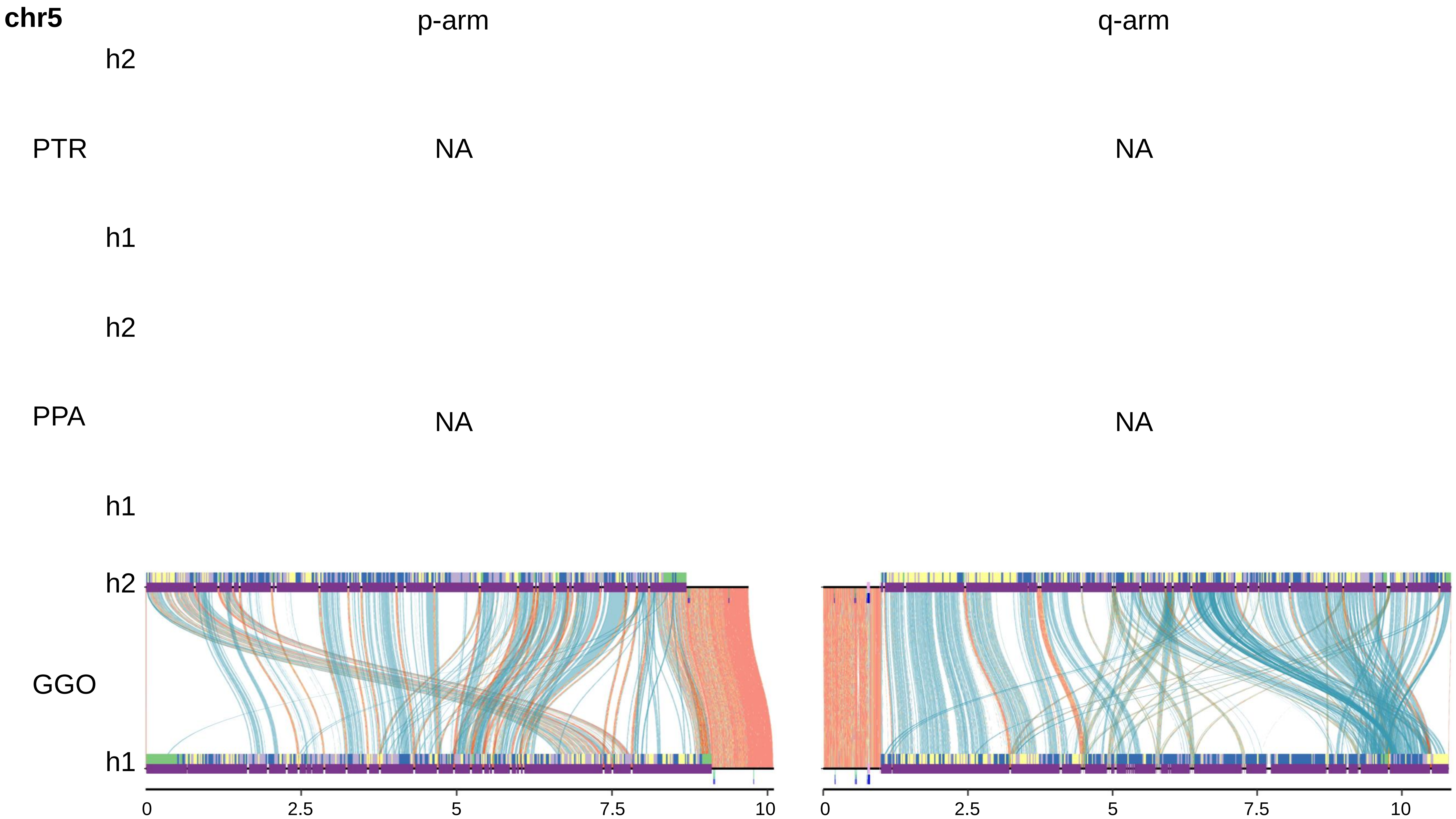
chr5
p-arm
q-arm
h2
PTR
NA
NA
h1
h2
PPA
NA
NA
h1
h2
GGO
h1
0
2.5
5
7.5
10
0
2.5
5
7.5
10

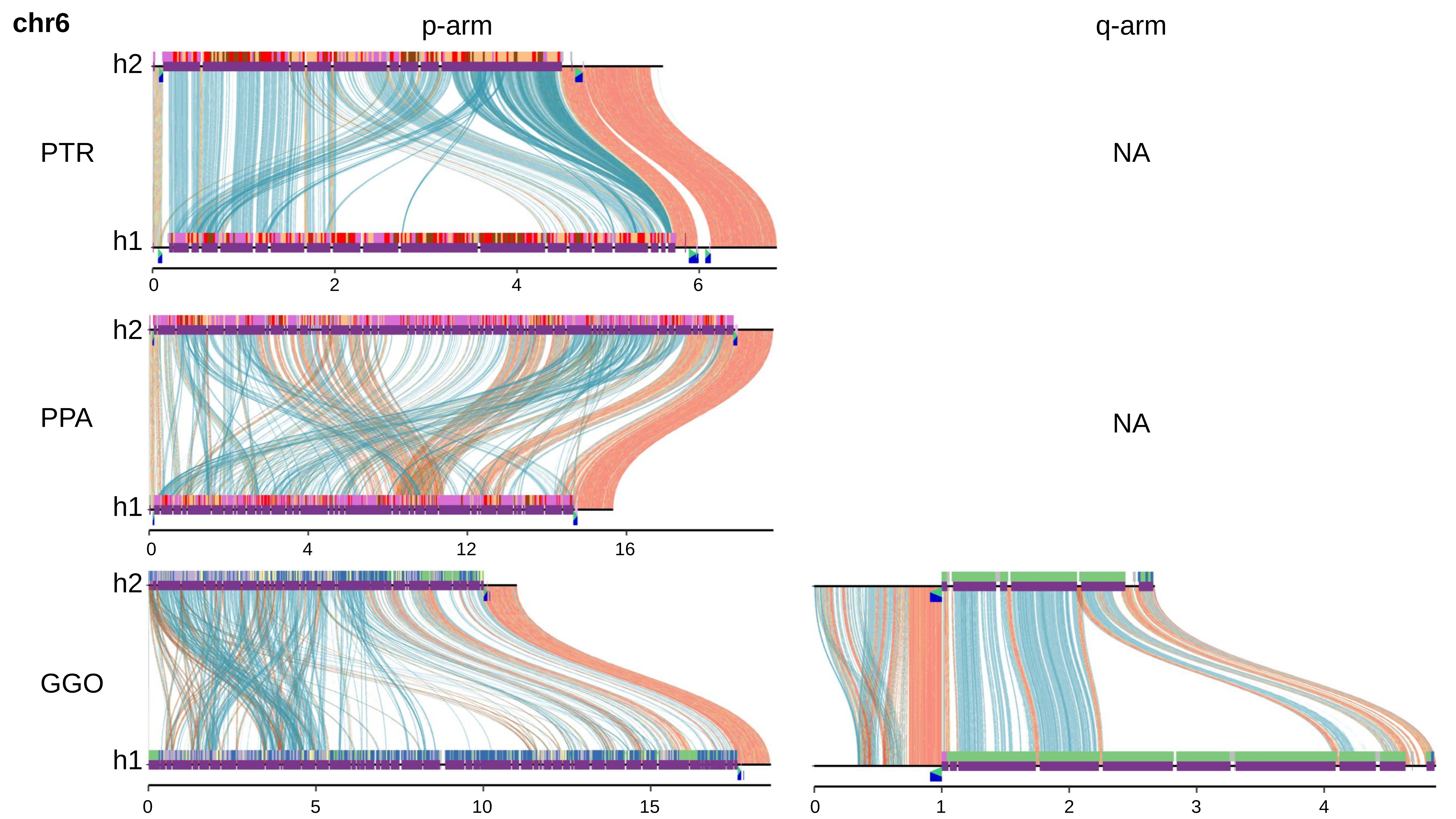
chr6
p-arm
q-arm
PTR
PPA
GGO
h2
h1
NA
NA
0
2
4
6
0
4
12
16
0
5
10
15
0
1
2
3
4

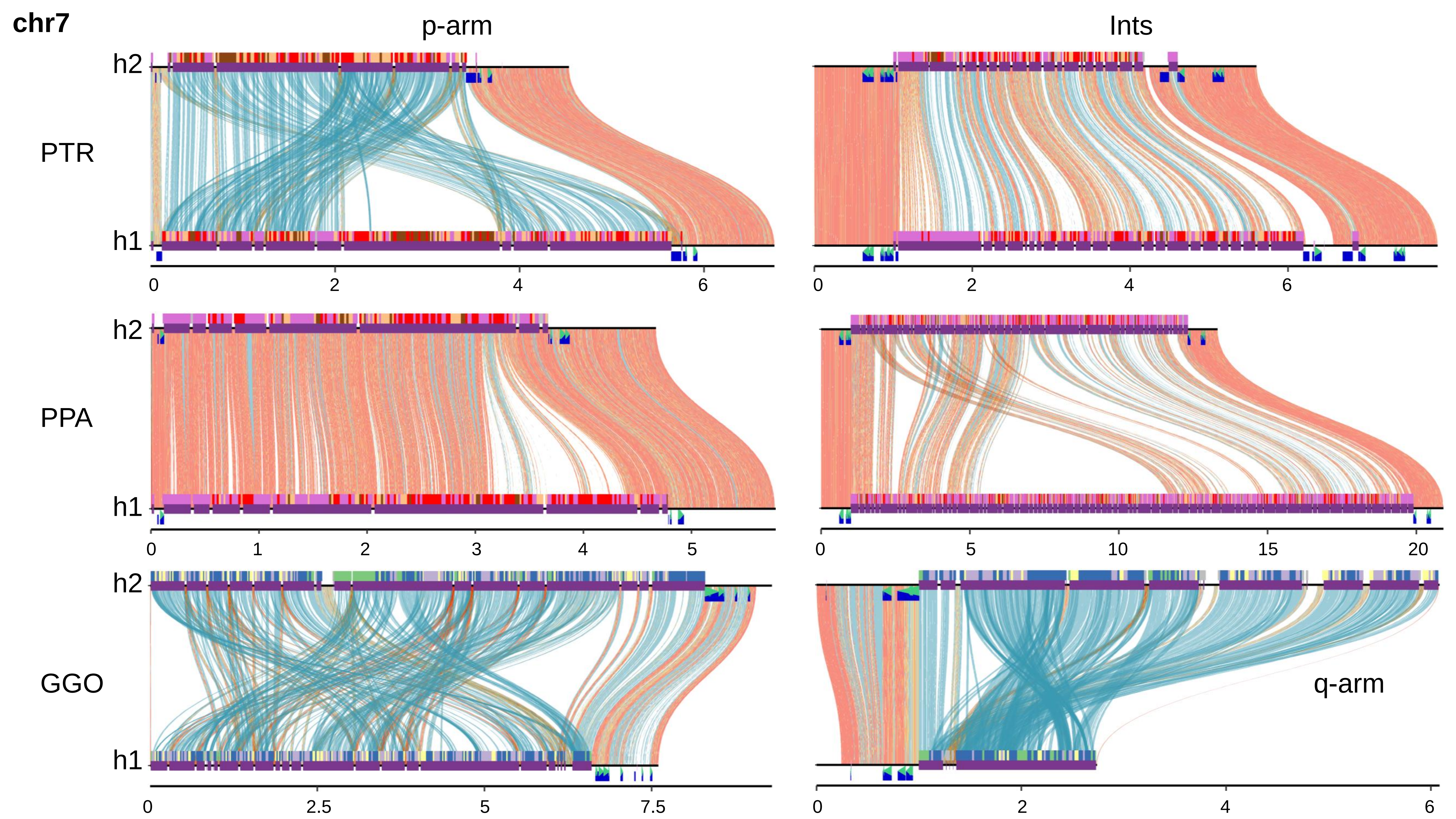

chr7
p-arm
lnts
h2
h1
PTR
PPA
GGO
q-arm
0
2
4
6
1
3
5
10
15
20
2.5
7.5

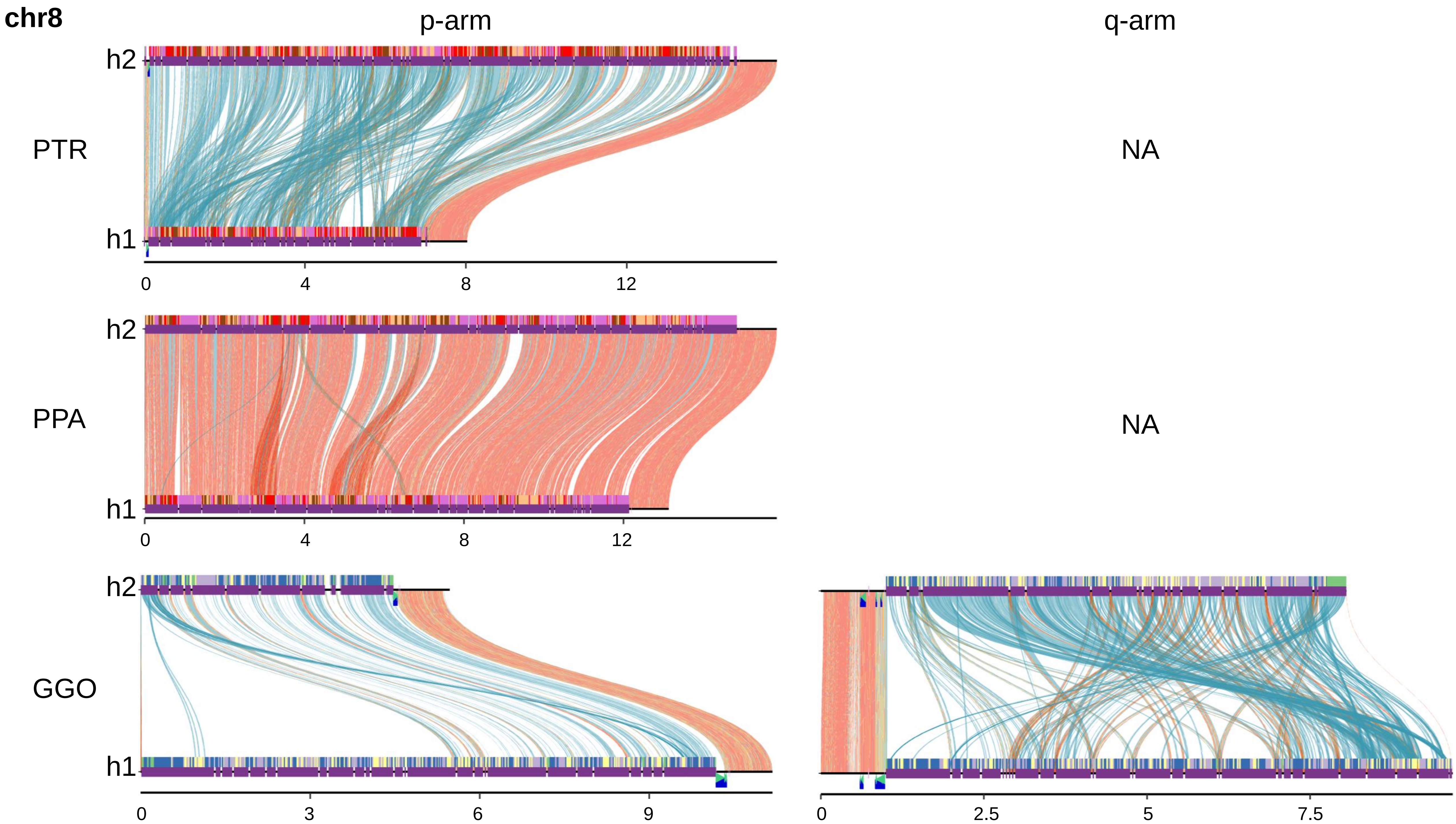
chr8
p-arm
q-arm
h2
h1
PTR
NA
0
4
8
12
h2
h1
PPA
NA
0
4
8
12
h2
h1
GGO
0
3
6
9
0
2.5
5
7.5

chr9

p-arm

q-arm

h2

PTR NA

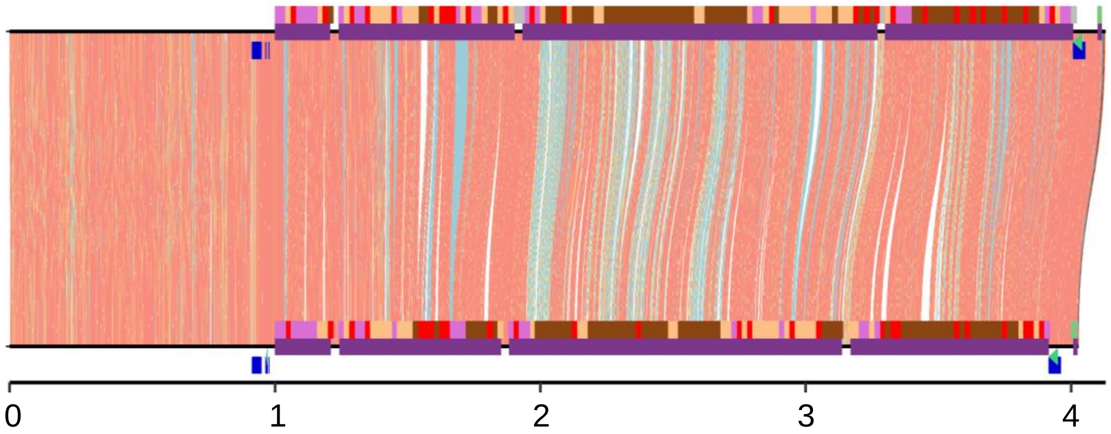

h1

h2

PPA NA NA

h1

h2

GGO NA NA

h1

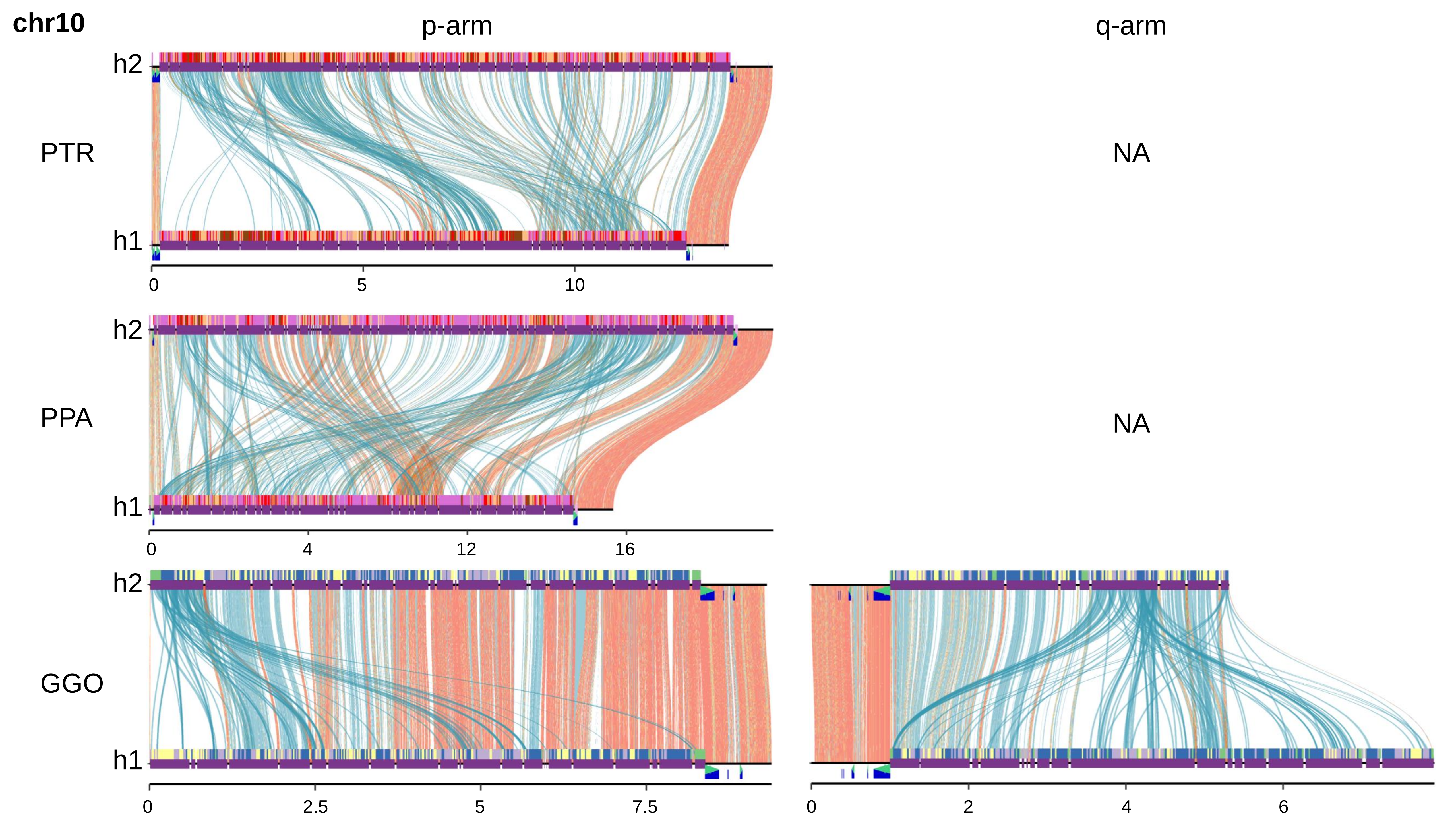
chr10
p-arm
q-arm
h2
h1
PTR
NA
0
5
10
h2
h1
PPA
NA
0
4
12
16
h2
h1
GGO
0
2.5
5
7.5
0
2
4
6

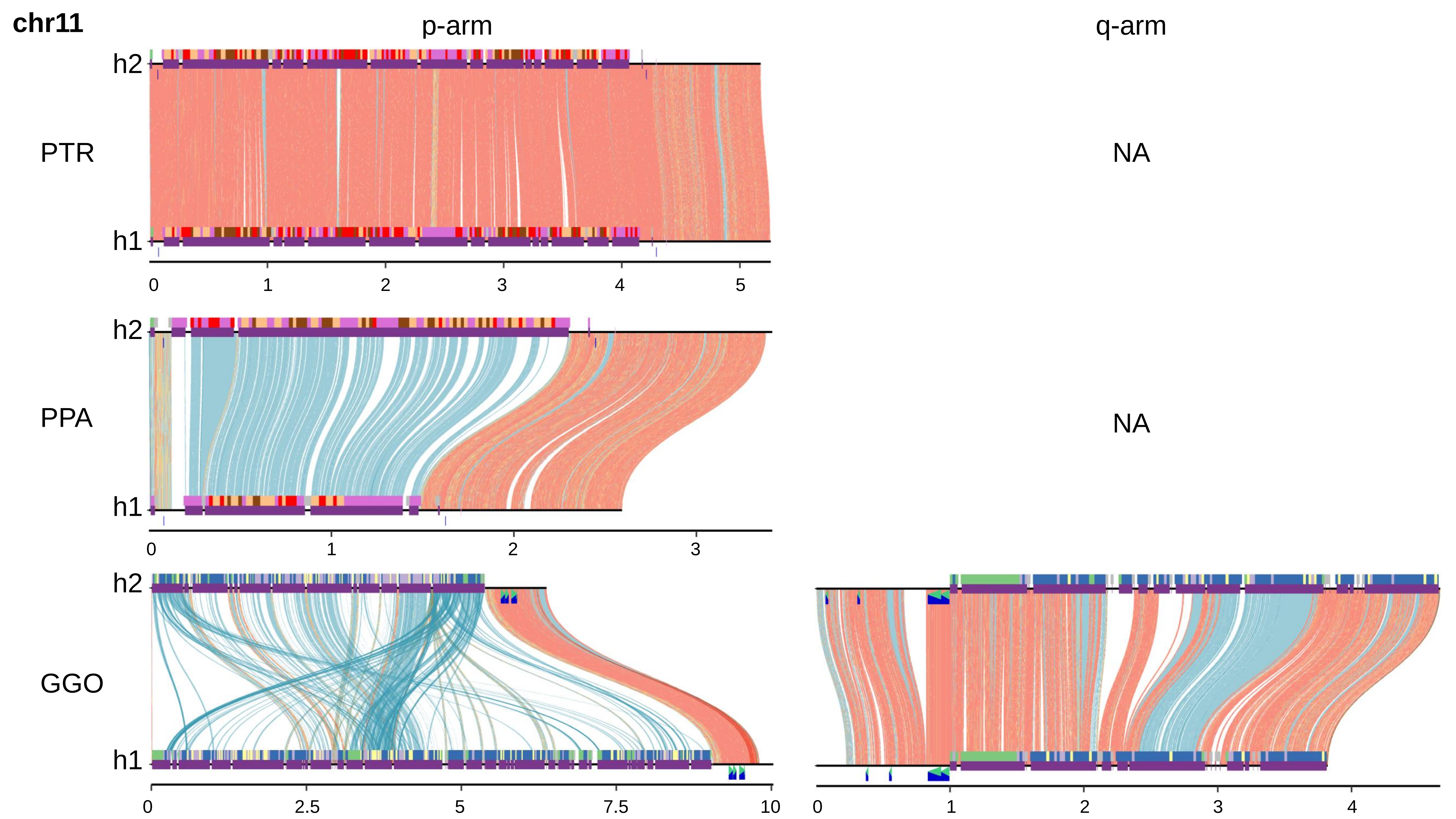
chr11
p-arm
q-arm
h2
h1
PTR
NA
0
1
2
3
4
5
h2
h1
PPA
NA
0
1
2
3
h2
h1
GGO
0
2.5
5
7.5
10
0
1
2
3
4

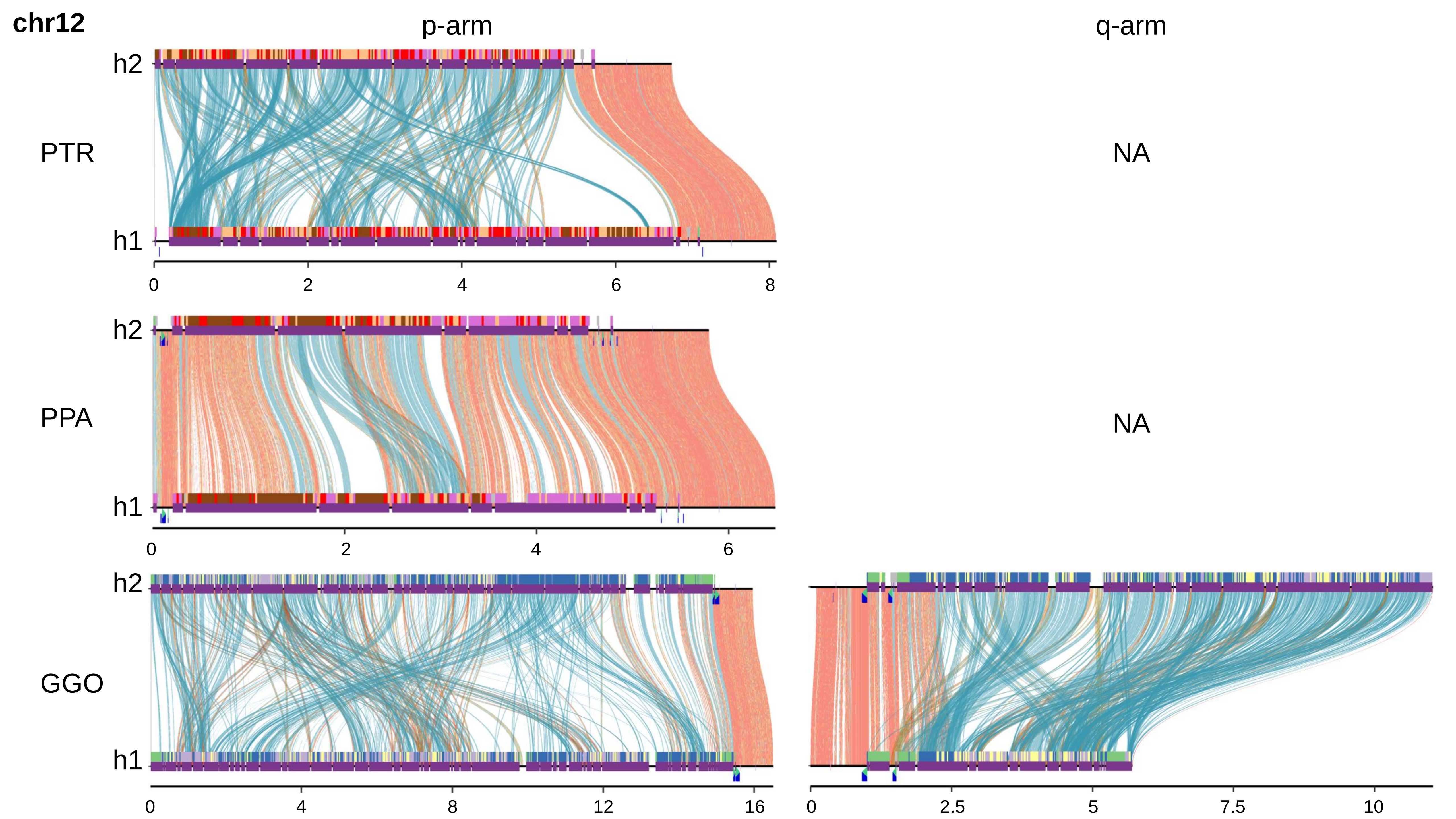
chr12
p-arm
q-arm
PTR
NA
PPA
NA
GGO
h2
h1
0
2
4
6
8
0
2
4
6
0
4
8
12
16
0
2.5
5
7.5
10

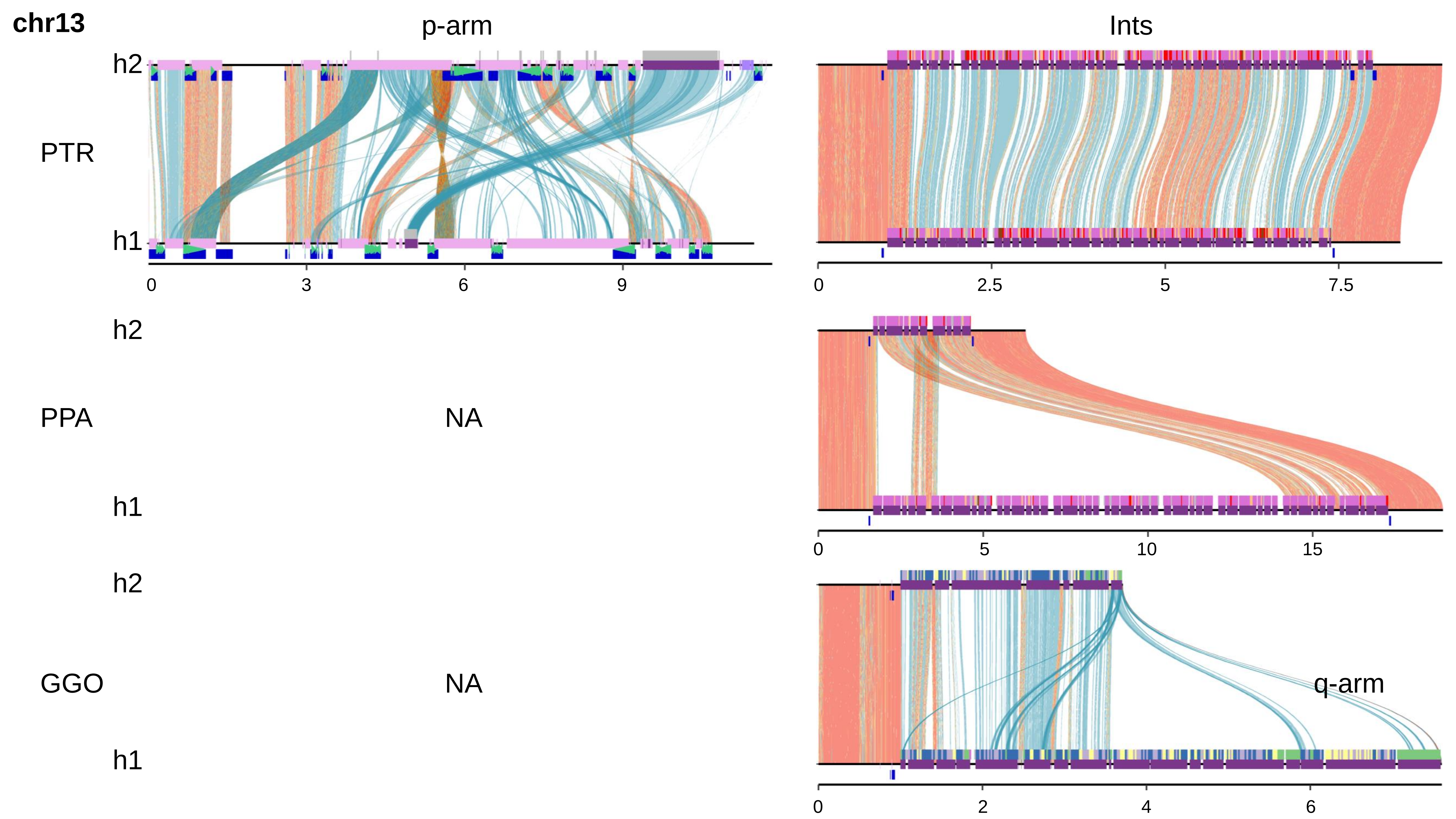
chr13
p-arm
lnts
h2
h1
PTR
0
3
6
9
0
2.5
5
7.5
h2
h1
PPA
NA
0
5
10
15
h2
h1
GGO
NA
q-arm
0
2
4
6

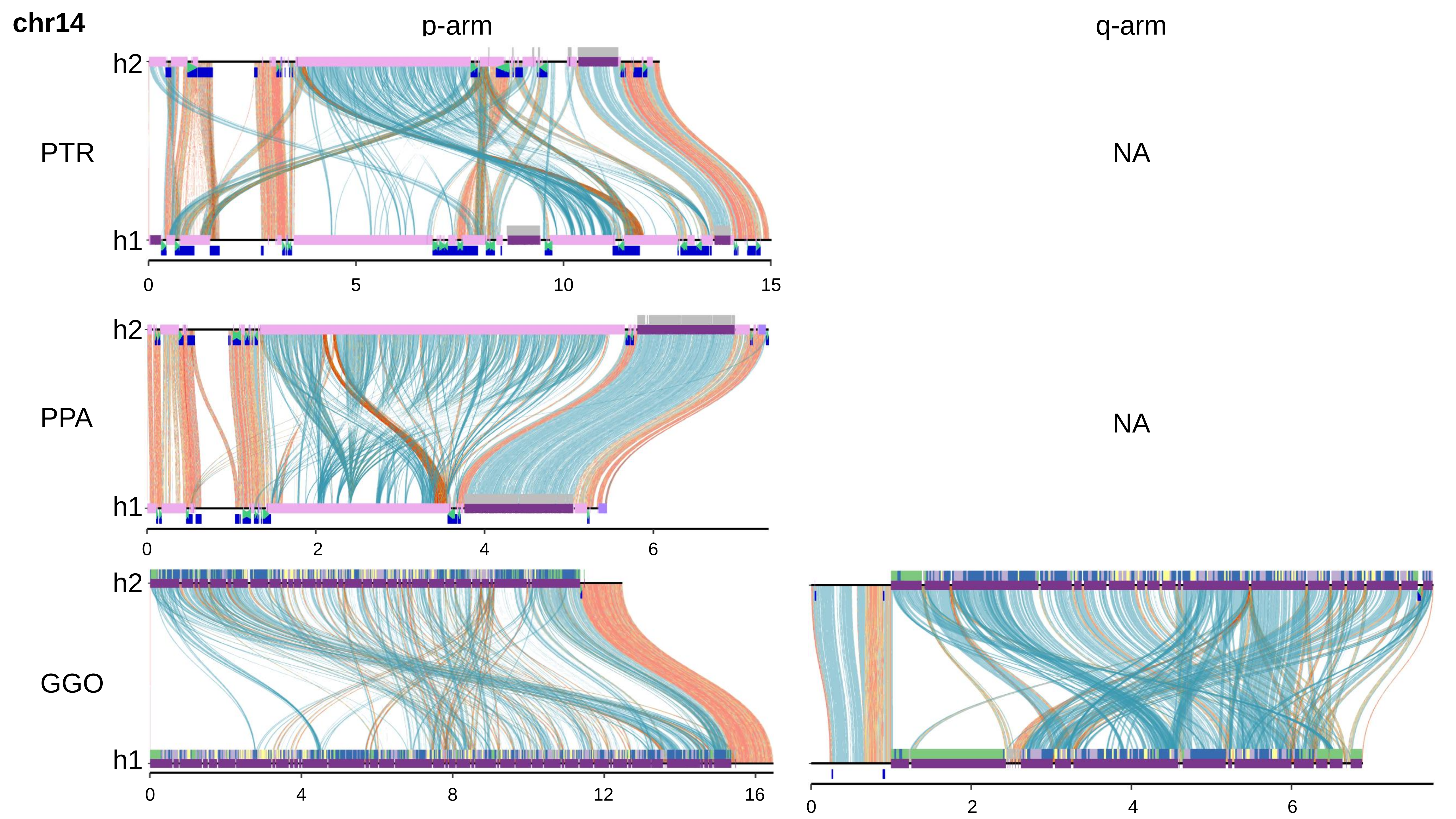
chr14
p-arm
q-arm
h2
h1
PTR
NA
0
5
10
15
h2
h1
PPA
NA
0
2
4
6
h2
h1
GGO
0
4
8
12
16
0
2
4
6

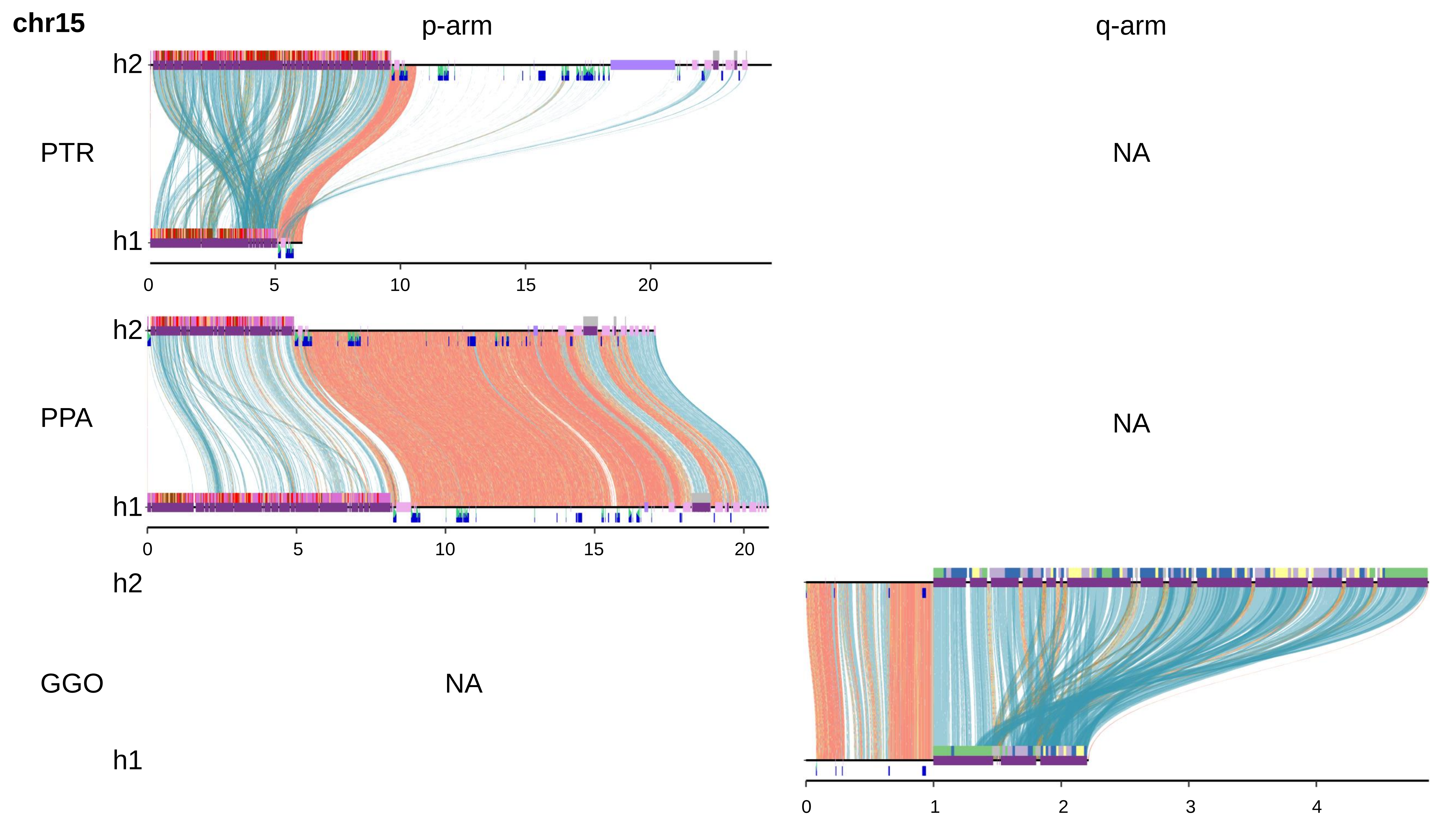
chr15
p-arm
q-arm
h2
h1
PTR
NA
0
5
10
15
20
h2
h1
PPA
NA
0
5
10
15
20
h2
h1
GGO
NA
0
1
2
3
4

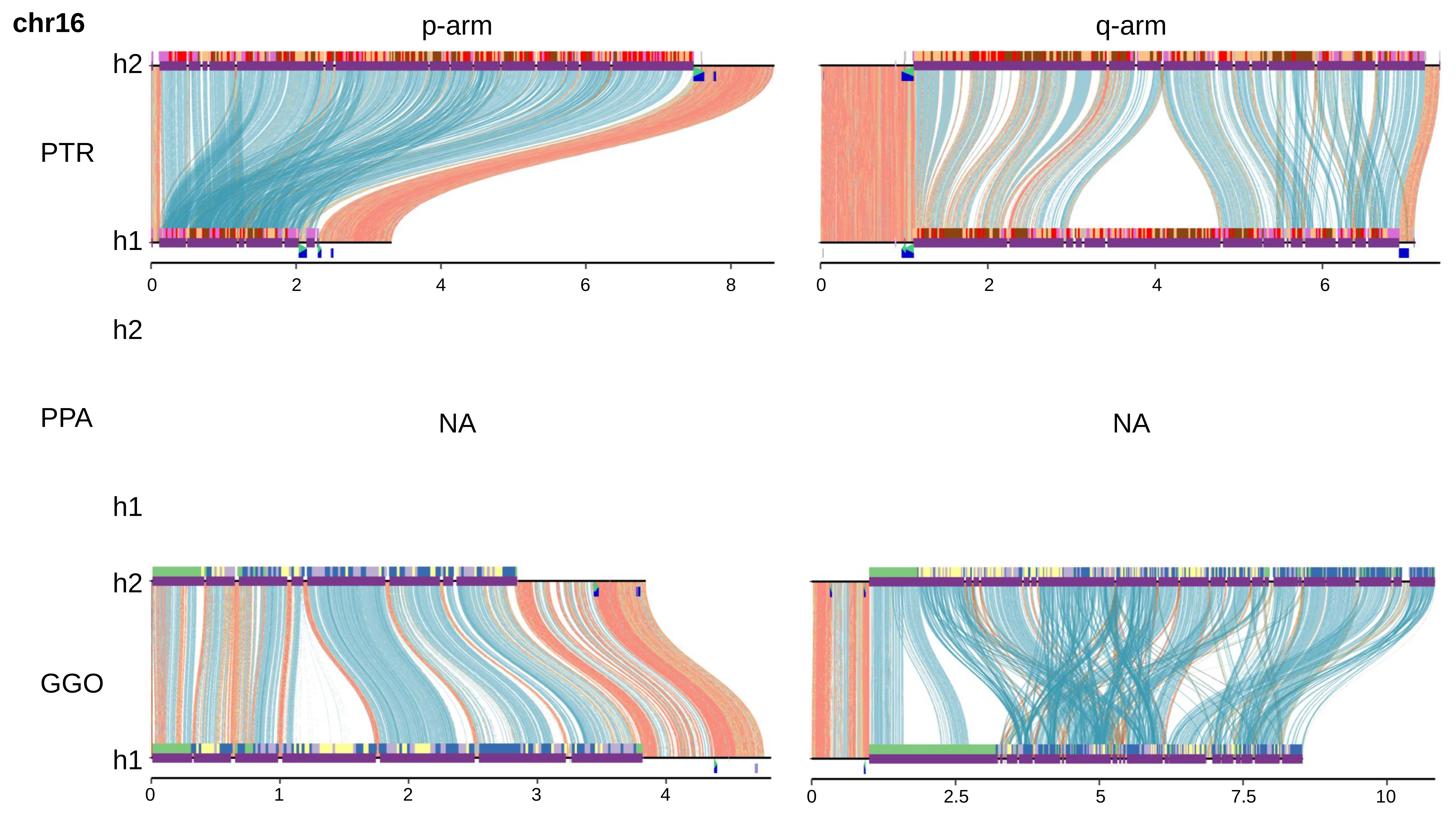
chr16
p-arm
q-arm
PTR
PPA
GGO
h2
h1
h2
h1
h2
h1
NA
NA
0
2
4
6
8
0
2
4
6
0
1
2
3
4
0
2.5
5
7.5
10

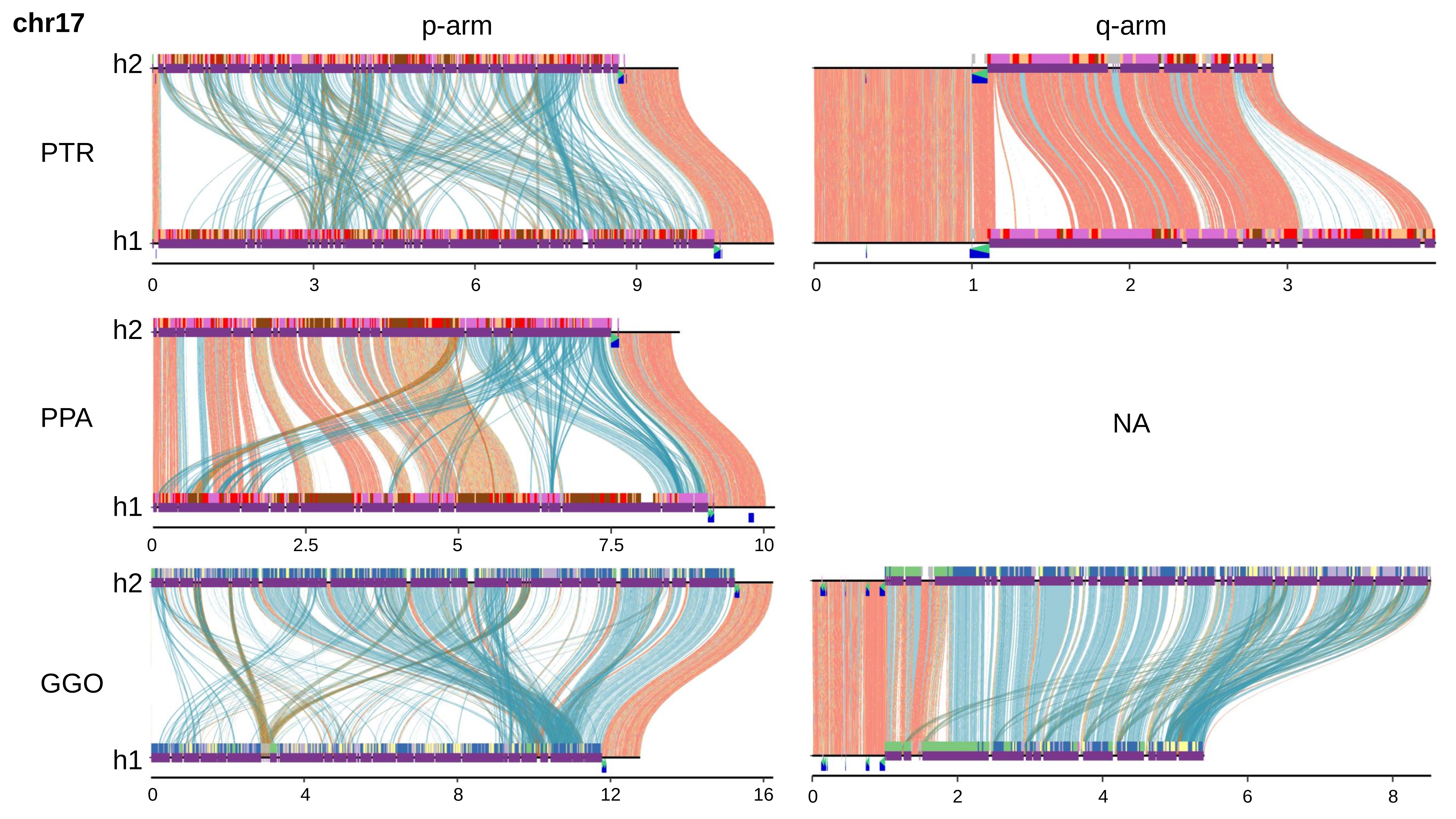

chr17
p-arm
q-arm
h2
h1
PTR
PPA
GGO
NA
0
3
6
9
1
2
2.5
5
7.5
10
4
8
12
16

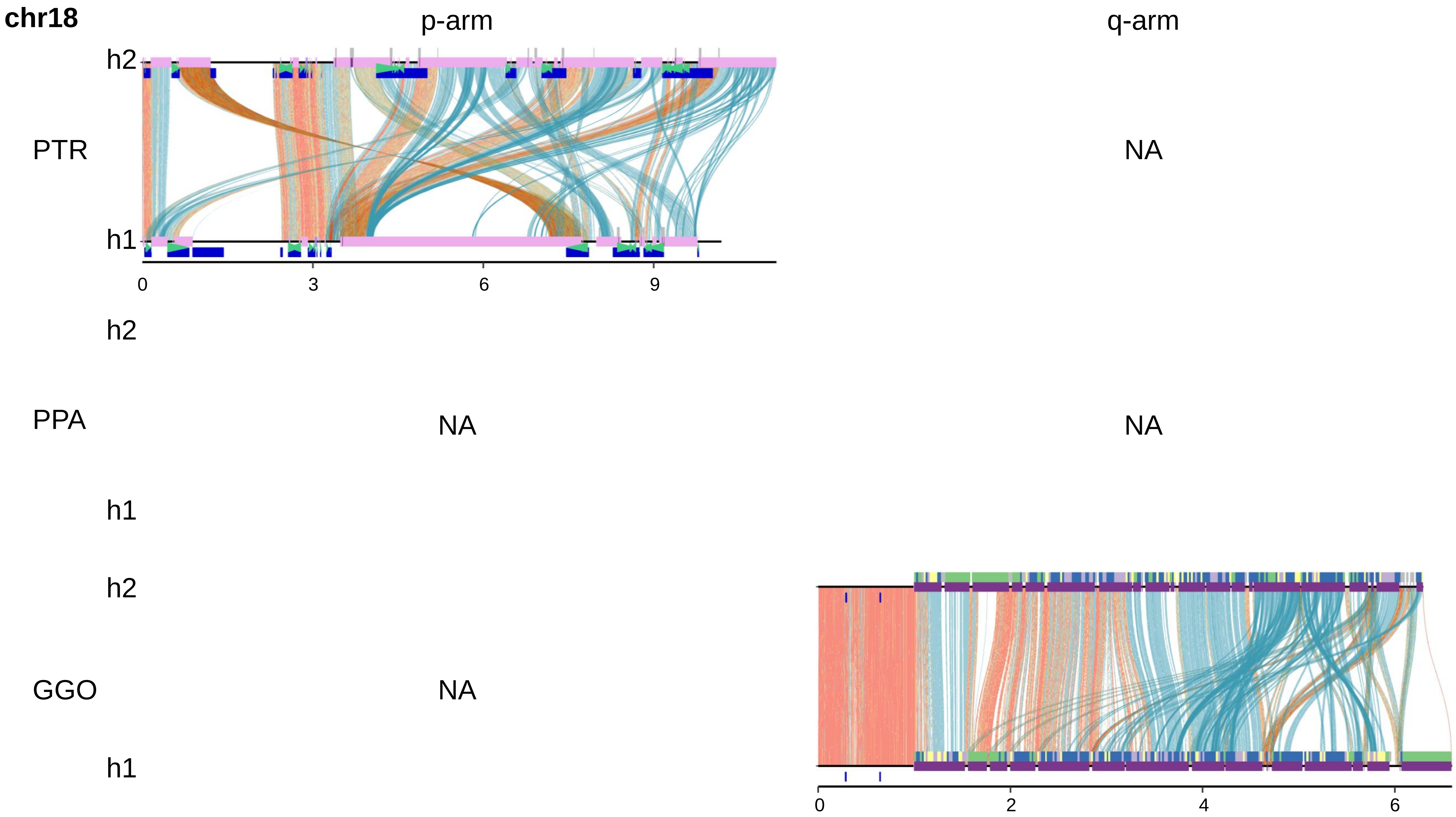

chr18
p-arm
q-arm
h2
PTR
h1
0
3
6
9
NA
h2
PPA
NA
NA
h1
h2
GGO
NA
h1
0
2
4
6

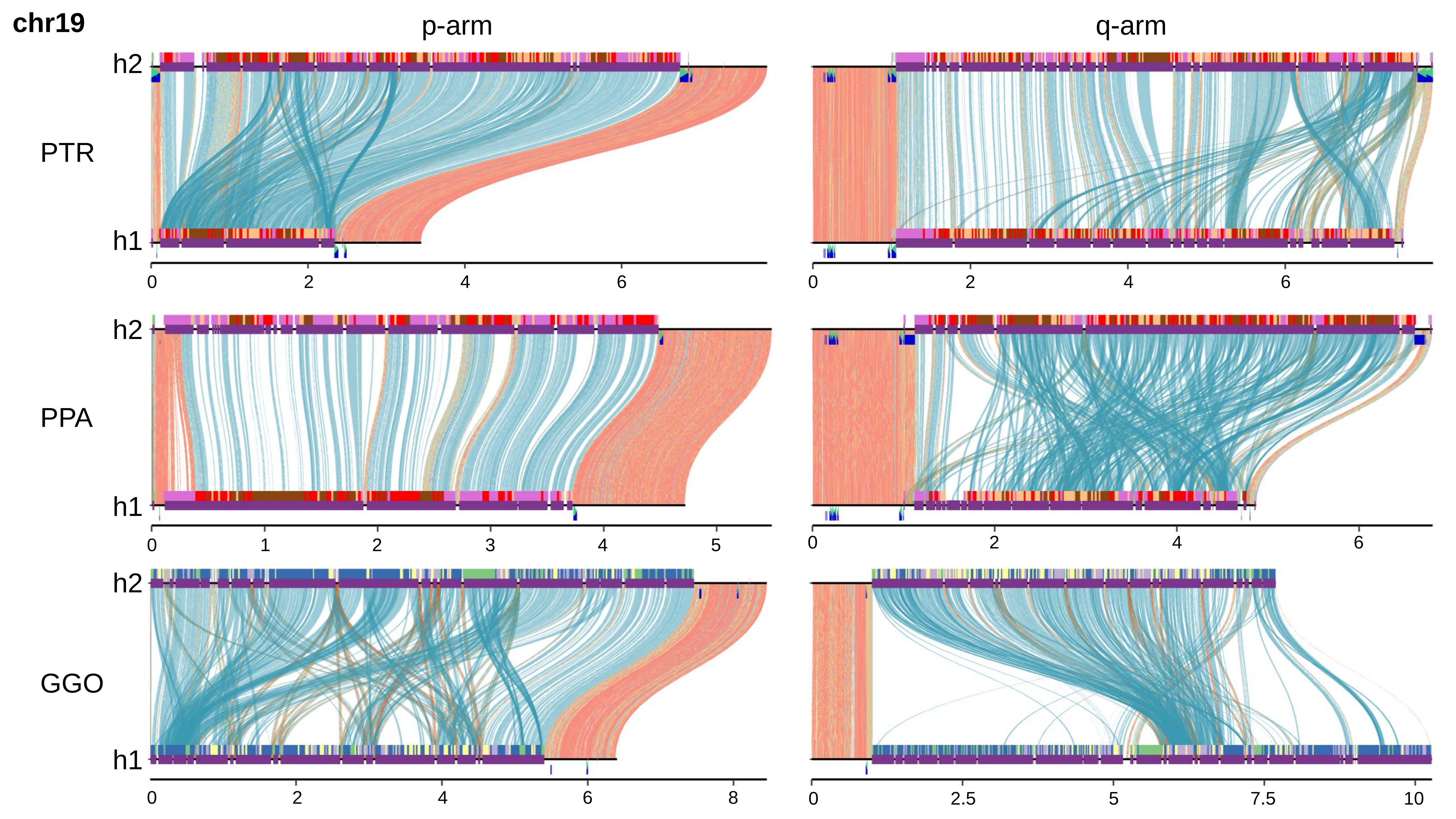

chr19
p-arm
q-arm
h2
h1
PTR
PPA
GGO
0
2
4
6
1
3
5
8
2.5
5
7.5
10

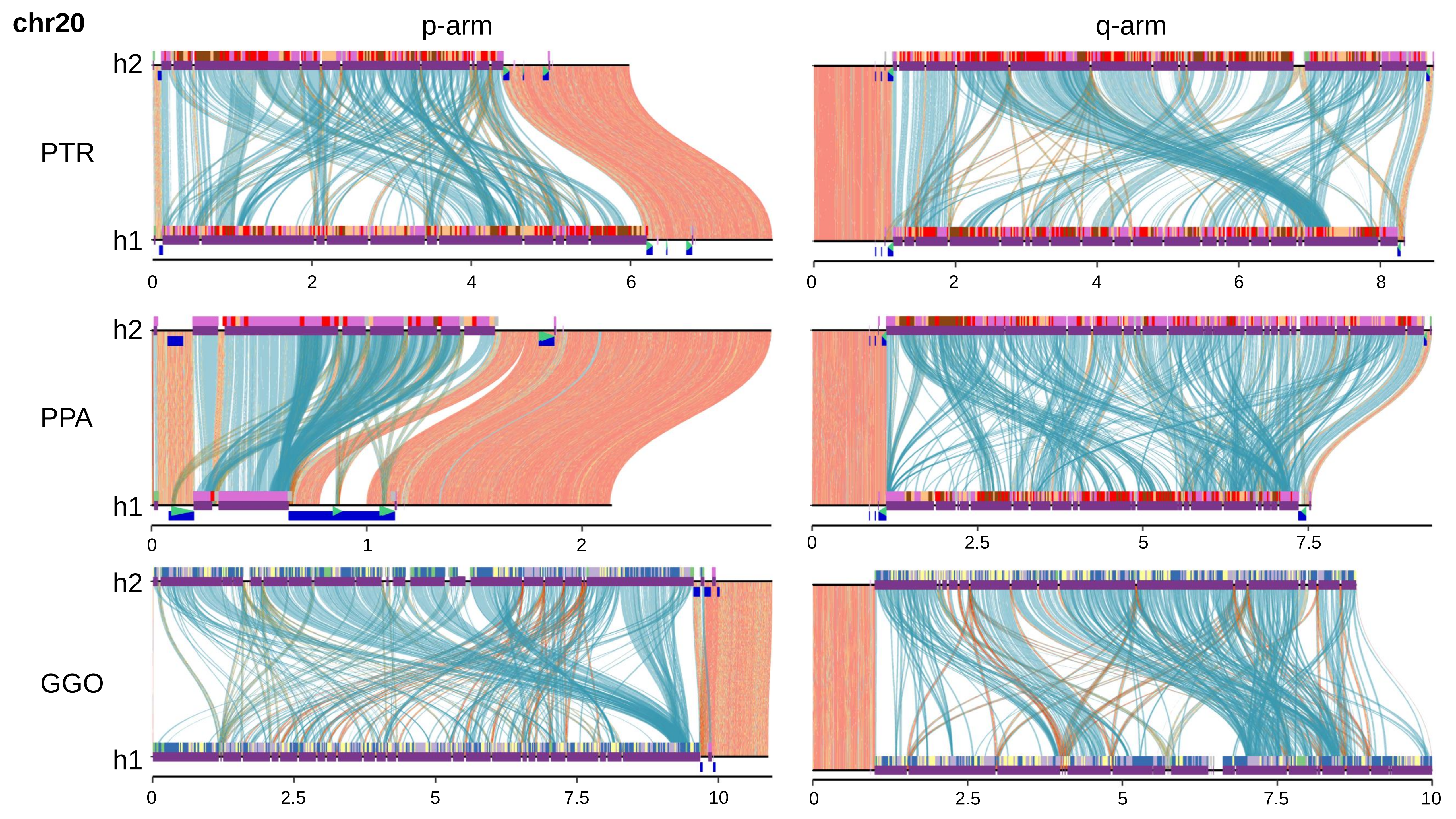

chr20
p-arm
q-arm
PTR
PPA
GGO
h2
h1
0
2
4
6
8
0
1
2
2.5
5
7.5
10

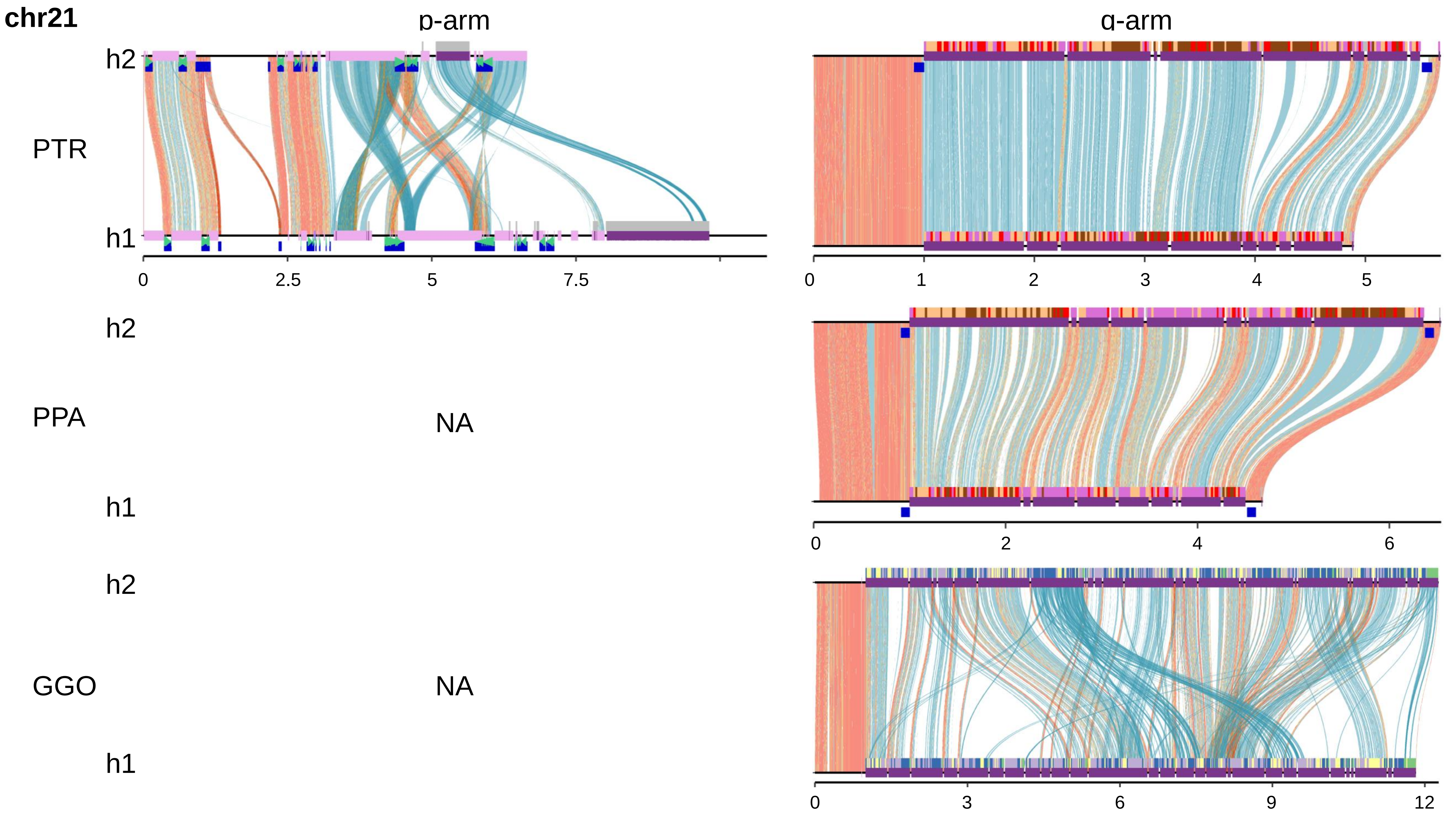
chr21
p-arm
q-arm
PTR
PPA
GGO
h2
h1
NA
NA
0
2.5
5
7.5
0
1
2
3
4
5
0
2
4
6
0
3
6
9
12

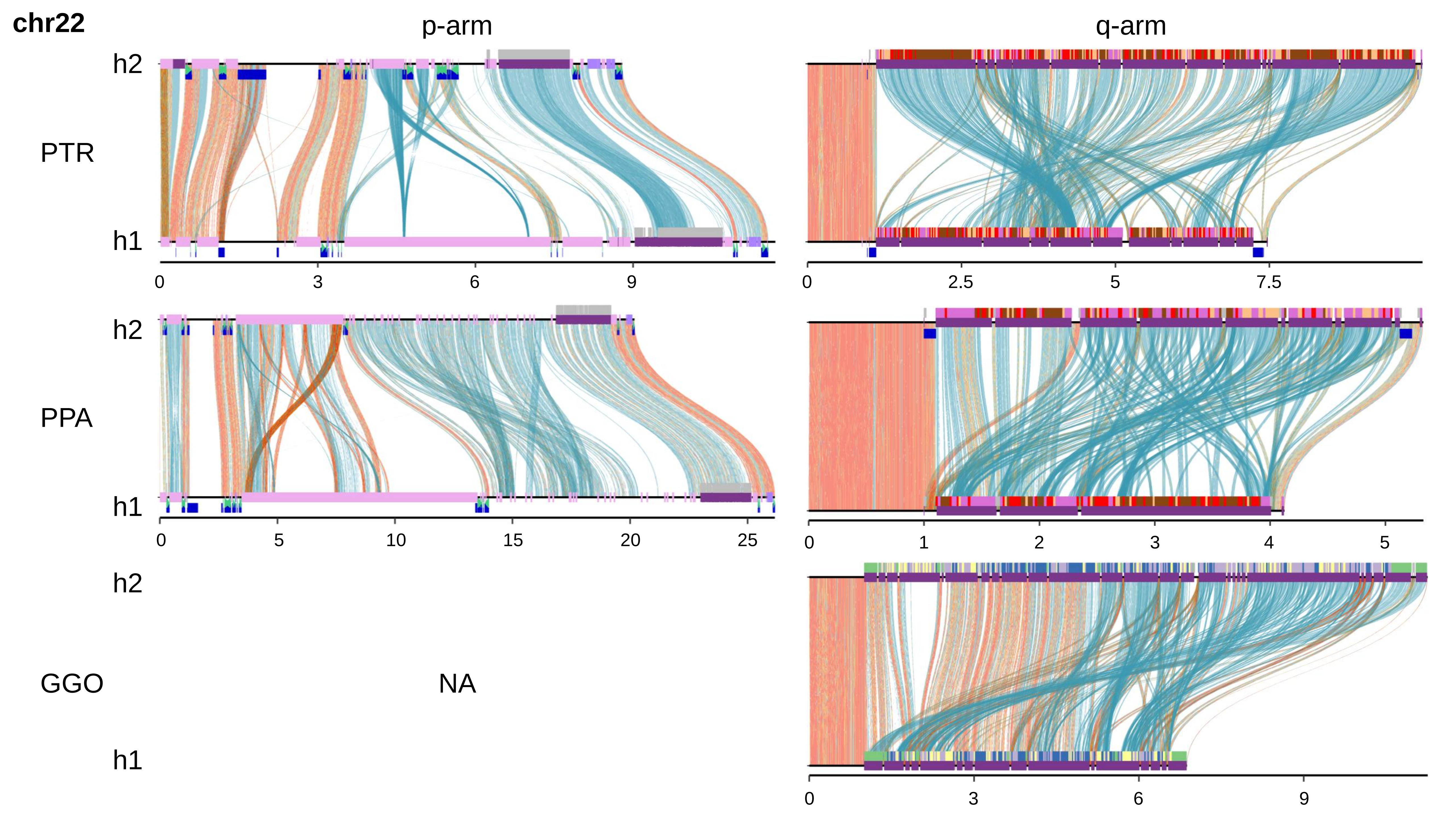

chr22
p-arm
q-arm
h2
h1
PTR
0
3
6
9
0
2.5
5
7.5
h2
h1
PPA
0
5
10
15
20
25
0
1
2
3
4
5
h2
h1
GGO
NA
0
3
6
9
